# Frailness and resilience of gene networks predicted by detection of co-occurring mutations via a stochastic perturbative approach

**DOI:** 10.1038/s41598-020-59036-w

**Published:** 2020-02-14

**Authors:** Matteo Bersanelli, Ettore Mosca, Luciano Milanesi, Armando Bazzani, Gastone Castellani

**Affiliations:** 1 Department of Physics and Astronomy, University of Bologna, Bologna, 40127 Italy; 2 National Institute for Nuclear Physics (INFN), Bologna, 40127 Italy; 3 Institute of Biomedical Technologies, National Research Council, Segrate, Milan, 20090 Italy

**Keywords:** Breast cancer, Stochastic modelling

## Abstract

In recent years complex networks have been identified as powerful mathematical frameworks for the adequate modeling of many applied problems in disparate research fields. Assuming a Master Equation (ME) modeling the exchange of information within the network, we set up a perturbative approach in order to investigate how node alterations impact on the network information flow. The main assumption of the perturbed ME (pME) model is that the simultaneous presence of multiple node alterations causes more or less intense network frailties depending on the specific features of the perturbation. In this perspective the collective behavior of a set of molecular alterations on a gene network is a particularly adapt scenario for a first application of the proposed method, since most diseases are neither related to a single mutation nor to an established set of molecular alterations. Therefore, after characterizing the method numerically, we applied as a proof of principle the pME approach to breast cancer (BC) somatic mutation data downloaded from Cancer Genome Atlas (TCGA) database. For each patient we measured the network frailness of over 90 significant subnetworks of the protein-protein interaction network, where each perturbation was defined by patient-specific somatic mutations. Interestingly the frailness measures depend on the position of the alterations on the gene network more than on their amount, unlike most traditional enrichment scores. In particular low-degree mutations play an important role in causing high frailness measures. The potential applicability of the proposed method is wide and suggests future development in the control theory context.

## Introduction

Complex networks arising from increasing sources of relational data in fields ranging from finance to medicine are capturing the attention and the effort of many researchers. Novel network-based methods for the analysis of complex data are being heavily introduced in order to face specific issues in many different academic fields. For example, only in the biomedical context, new technologies for collecting data on the physical interactions among biomolecules are constantly updating the knowledge about molecular networks^1^. From such measurements many network-based data integration methods are being developed^2–25^. Molecular networks are models of the complex interactions among molecular entities within cells, providing a mathematical environment for the integrated analysis of one or more types of biological data. In the literature, algorithms that integrate molecular information and molecular networks are being proposed in several applications^2–10^, giving rise to the network biology and network medicine frameworks^26–28^.

Many network-based approaches in the systems biology context aim at molecular mechanism discovery, sample stratification and phenotype prediction^29^. The majority of network-based methods recently introduced are characterized by an implicit *enrichment paradigm*: the tacit assumption of such approaches, independently from the specific goal or technique, is that those systems (e.g. molecular pathways, gene networks) mostly enriched with biological signal will be considered as the most relevant part of the results, like for example the whole class of methods carrying out over representation analysis.

In this work we introduce a new network based method that, as we will illustrate, partially deviates from the *enrichment paradigm*. We investigated how the combined presence of altered nodes perturbs the information flow within a given network, where the probability that each couple of nodes exchange information was modeled by a perturbed master equation (pME). We used the general term “information” in order to underline that the stochastic process described by the ME implies an exchange of an ideal substance between network nodes. In this work we defined the pME model in a general setting, while we considered gene networks and gene mutations in cancer, in order to build a straightforward application. In principle, depending on the context and on the specific application (e.g. biochemical, transportation, or financial networks) the pME may imply more complex and ad hoc formulations, including for instance chemical reactions between network nodes, transportation or transaction dynamics.

The proposed method aims at quantifying how much a specific network is perturbed by the simultaneous presence of altered nodes, in terms of how much it deviates the trajectories of information flow with respect to the non-perturbed state of the network. From the comparison between the stationary distributions of the ME respectively with and without the perturbation we defined the frailness of a perturbed network.

Network frailness is intended as opposite concept to network resilience. Resilience, a concept developed within dynamical systems theory, is becoming important in biology and medicine^30^, but also in other fields, such as ecological and financial networks. Resilience is defined as the capacity of a system to resist to perturbation and to recover quickly, whereas frailness has the opposite meaning. Network frailness, is conceptually similar to frailty, as used in aging research, where indicates the individual vulnerability to poor outcomes. Here we use the term frailness in a broader sense, as the reduced capacity of an organism (at multiple level) to deal with an external perturbation.

Interestingly, network frailness presents a connection with the controllability of a network defined in a classical control theory context^31^. Control theory asks how to influence the behavior of a dynamical system with appropriately chosen inputs so that the system’s output follows a desired trajectory or final state after a given time interval. In recent years many concepts defined by control theory were successfully inherited by network theory^32–35^ as a complex network naturally defines the transition matrix of a dynamical system. The pME model introduces the possibility to set up an alternative control theory formulation where, unlike classic control theory, the system’s inputs exchange information with the environment, as we will discuss further. Such approach would allow to characterize the totality of altered nodes combinations leading the network dynamics toward a diverging behavior of the pME. The advantage of control is the possibility to determine the control ability of any set of nodes indipendently from the known node alterations. In particular, the results described here are close to the concept of *control range*^36^, which quantifies the responsibility of a node in controlling a network together with other driver nodes. For simplicity in this work we focused on the presentation of the pME model and its implications, leaving the rigorous definition of the pME model from a control theory perspective to future work.

**Figure 1:**
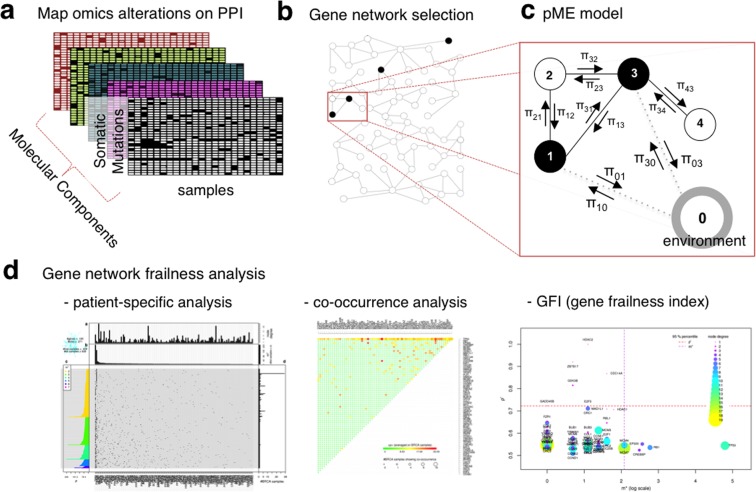
Overview of perturbative approach application to biological data. (**a**) The omic information is mapped on the interactome (PPI). We focused on somatic mutations (SM) but the same analysis is performable with different types of omic information. (**b**) A significant gene network is considered for the analysis. (**c**) Application of perturbed ME model to the selected gene network. (**d**) For each sample we measure the frailness of the molecular alterations on the target network; the analysis is carried out considering patient-specific insights, co-occurrence analysis and Gene Frailness Index (GFI), in order to capture synthetic insights.

In order to show the applicability of the pME in the biomedical context, we presented a pipeline for biological networks frailness analysis as illustrated in Fig. 1. Considering any omic database (1a) (e.g. molecular information on the rows and samples on the columns), the molecular alterations define the query nodes that are mapped on the protein-protein interaction network, from which a biologically significant gene subnetwork is selected (1b) similarly to Vinayagam *et al*.^37^. On the target gene network each sample’s altered nodes are then modeled according to the pME (1c) in order to set up the gene network frailness analysis (1d). Considering the analysis for varying samples and gene networks we finally investigated the gene-gene co-occurrences causing frail gene networks and proposed the Gene Frailness Index (GFI), a 2-dimensional bioinformatic score accounting for the responsibility of each molecular alteration in causing network frailness in a given disease (1d).

As a proof of principle we applied the proposed method to somatic mutations (SM) detected in breast cancer (BC) samples^38^ using biologically significant target gene networks. Such target gene networks are defined by jointly considering KEGG human pathways^39^ and the HuRI protein-protein interaction network^40^.

The code developed for the analysis is available upon request.

## Methods

### General formulation of the pME

We considered an M-nodes connected network that in principle can be also weighted and directed. For simplicity, we developed the model considering an undirected and unweighted network, but the model can be easily extended to more complex set ups. On the given network structure we defined a stochastic process modeled by a master equation (ME), describing at a microscopic level the jumps of ideal particles along the existing network edges, while at a macroscopic level, the particle’s flow between network nodes can be interpreted as the evolution of an ideal substance on the network that we summarized with the general term “information”. In this section we introduce the pME from a general perspective, the specific setting used for the application to biological data is described in next sections.

Let *π*_*kj*_ be the information transition rate between node *j* and node *k* and let $\overrightarrow{p}(t)$ be the M-dimensional state of the network at time *t*, where the k-th entry *p*_*k*_(*t*) represents the average probability to find that node *k* is actively exchanging information at time *t*; the ME reads

1 $$\dot{\overrightarrow{p}}+L\overrightarrow{p}=0$$

where we use the Laplacian matrix *L* as suggested in the work of Mirzaev and Gunawardena^41^. The Laplacian matrix is defined as $L=D-\Pi$ where *D* is a diagonal matrix containing the loss probability of each node to any other node $({d}_{kk}=\sum _{j}\,{\pi }_{jk})$ while Π is the information transition matrix. Equation (1) corresponds to the continuity equation for the probability distribution with the constraint $\sum _{k}\,{p}_{k}(t)=1$. Its stationary solution ${\overrightarrow{p}}_{s}$ corresponds to the kernel of the matrix *L*. Given Eq. (1), we explicitly consider the case when the Laplacian matrix *L* is self-adjoint with respect to a given scalar product, that is equivalent to a detailed balance condition for the stationary state of the ME. Under such assumption, *L* has all positive eigenvalues except the first one which is zero, so that the stationary state is unique and attractive.

**Figure 2:**
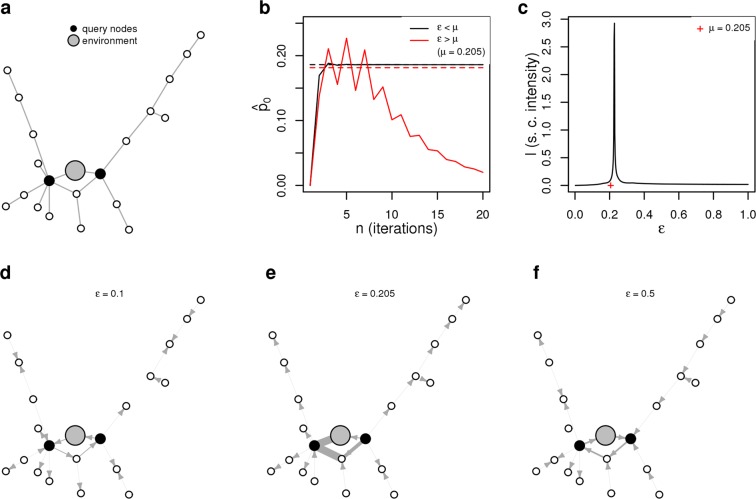
Critical frailness threshold and steady flow currents. (**a**) We perturbed a fully connected 20-nodes synthetic network with the query nodes colored in black by defining new connections to the environment node colored in gray. (**b**) Behavior of the 0-th component of the iterative scheme ${\hat{p}}_{0}$ with input values smaller (black line) and bigger (red line) with respect to the critical threshold $\mu$. (**c**) Non-linearity in stationary current intensity $I$ in correspondence of the critical frailness threshold $\mu$. (**d**) Steady flow probability currents with input below the critical value. (**e**) Steady flow probability currents with input corresponding to the critical value. (**f**) Steady flow probability currents with input above the critical value.

We now consider the presence of a network perturbation as an external node (0) playing the role of the environment, exchanging information with the target network *only* through the interaction with the altered nodes (query nodes) (Fig. 2a). Information can be introduced from the environment into the network and can return back into the environment according to the novel connections from (*π*_*k*0_) and to (*π*_0*k*_) the environment, regulated by an intensity parameter *ε*. We therefore consider an extended (M + 1 dimensional) master equation

2 $$\dot{\overrightarrow{p}}+({L}_{0}+\Delta {L}_{\varepsilon })\overrightarrow{p}=0$$

where *L*_0_ is the extension of the Laplacian matrix *L* where the environment node is decoupled from the existing network, while the network perturbation is encoded in the Laplacian perturbation matrix $\Delta {L}_{\varepsilon }$, where we underline the dependence of the perturbation from the intensity parameter $\varepsilon$ (See Supplementary Information for details). Once fixed the perturbation intensity $\varepsilon$, Eq. (2) has a unique stationary solution ${\overrightarrow{p}}_{s}^{\ast }$. We do not solve the system (2) directly since we are interested in finding an intrinsic measure to quantify how much a network perturbation $\Delta {L}_{\varepsilon }$ deviates the stationary distribution (${\overrightarrow{p}}_{s}^{\ast }$) away from the unperturbed stationary distribution (${\overrightarrow{p}}_{s}$).

The self-adjoint assumption about matrix *L* with respect to a scalar product implies that, given any couple of M-dimensional vectors $\overrightarrow{u},\overrightarrow{w}$

3 $$\overrightarrow{u}\cdot L\overrightarrow{w}=L\overrightarrow{w}\cdot \overrightarrow{u}$$

where $\overrightarrow{u}\cdot \overrightarrow{w}:\,=\sum _{j,k}\,{u}_{j}{g}_{jk}{w}_{k}$ for a metric matrix *G*. It is straightforward to notice that all the eigenvalues of *L* are real and if ${\overrightarrow{v}}_{1},{\overrightarrow{v}}_{2}$ are two eigenvectors of *L* of eigenvalues respectively ${\lambda }_{1},{\lambda }_{2}$ (${\lambda }_{1}\ne {\lambda }_{2}$), their scalar product is null. This remark allows to find the orthogonal base of eigenvectors $\{{\overrightarrow{v}}_{1},{\overrightarrow{v}}_{2},\cdots ,{\overrightarrow{v}}_{M}\}$. Such a base can be naturally extended as we add the enviroment node to the model since the matrix *L*_0_ has a two-dimensional kernel $\langle {\overrightarrow{e}}_{0},{\overrightarrow{v}}_{1}\rangle$ where ${\overrightarrow{v}}_{1}={(0,{\overrightarrow{p}}_{s})}^{T}$ and ${\overrightarrow{e}}_{0}$ is the vector with 1 in the 0-th position and 0 elsewhere completing the orthogonal base of eigenvectors of matrix L. Omitting the vector notation and the subscript *ε* in the perturbation matrix for simplicity, we look for the stationary distribution of the perturbed system in the in the form

4 $${p}_{s}^{\ast }=\hat{p}+{\alpha }_{0}{p}_{s}+{e}_{0}$$

where $\hat{p}$ is orthogonal to both ${p}_{s}$ and ${e}_{0}$, and therefore is generated only by the remaining M-1 orthogonal eigenvectors. After some algebra we define the recurrence

5a $${L}_{0}\,{\hat{p}}_{n}=-\,(I-\Pi ){\hat{p}}_{n-1}{\alpha }_{n-1}(I-\Pi )\Delta L{p}_{s}+(I-\Pi )\Delta L{e}_{0}$$

5b $${\alpha }_{n}{p}_{s}\cdot \Delta L{p}_{s}=-\,{p}_{s}\cdot \Delta L{\hat{p}}_{n}-{p}_{s}\Delta L{e}_{0}$$

5c $${\alpha }_{n}{e}_{0}\cdot \Delta L{p}_{s}=-\,{e}_{0}\cdot \Delta L{\hat{p}}_{n}-{e}_{0}\Delta L{e}_{0}$$

where Π is the projection on the two-dimensional kernel of ${L}_{0}$. Equations (5b) and (5c) have to be linearly dependent so it is sufficient to consider a single equation. Let now use the extended orthogonal base to decompose ${\hat{p}}_{n}$

6 $${\hat{p}}_{n}=\sum _{k > 1}\,{c}_{n,k}{v}_{k}$$

Substituting Eq. (6) into Eqs. (5a, 5b), projecting on each eigenspace and recalling that ${L}_{0}{v}_{k}={\lambda }_{k}{v}_{k}$ by definition, we obtain the final iterative scheme

7a $$\lambda {c}_{n,k}=-\sum _{j > 1}\,{c}_{n-1,j}\bar{\Delta }{L}_{kj}-{\alpha }_{n-1}\bar{\Delta }{L}_{k1}-\bar{\Delta }{L}_{k0}$$

7b $${\alpha }_{n}=-\sum _{j > 1}\,{c}_{n,j}\bar{\Delta }{L}_{1j}/\bar{\Delta }{L}_{11}-\bar{\Delta }{L}_{10}/\bar{\Delta }{L}_{00}$$

where $\bar{\Delta }{L}_{kj}\,:={v}_{k}\cdot \Delta L{v}_{j}$ is the projection of the j-th perturbation component on the k-th one. When the sequence ${\hat{p}}_{n}$ converges with initial conditions

8 $${\hat{p}}_{0}=0,\,{\alpha }_{0}=1$$

we get the stationary solution of the perturbed system.

Iterative scheme (7a, b) is informative about the network dynamics since, once a set of query nodes is fixed, the iterative scheme converges or diverges according to the intensity $\varepsilon$ with which the network exchanges information with the environment. Indeed, such critical intensity threshold $\mu =\hat{\varepsilon }$ distinguishing between converging and diverging behaviors is retrievable numerically using a simple bisection method (see Fig. 2b).

From an analytic perspective, the convergence condition for the iterative scheme allows to compute the critical threshold *μ* as the biggest possible perturbation intensity $\varepsilon$ that satisfies the convergence condition:

9 $$\Vert \Delta {L{\prime} }_{\varepsilon }\Vert /{\lambda }_{F} < 1,$$

where ${\lambda }_{F}$ is the smallest non-zero eigenvalue of the Laplacian matrix *L* also called Fiedler number of the network, and $\Delta {L}_{\varepsilon }\text{'}$ is an appropriate transformation of the perturbation matrix that exploits the detailed balance condition $(\Delta {L{\prime} }_{kj}\,:={\bar{L}}_{kj}-\frac{{\bar{L}}_{k1}}{{\bar{L}}_{11}}{\bar{L}}_{1j})$ where we omitted the subscript $\varepsilon$ in matrix Δ*L*′. Given a distribution of query nodes on a target network, we observe a macroscopic change occurring in the pME in correspondence of the critical input value $\mu =\hat{\varepsilon }$, as we will further discuss in detail.

### Stationary information currents in the pME model

In the pME model, when the perturbation matrix $\Delta {L}_{\varepsilon }$ is fixed from a topological point of view, the critical threshold consists of a critical intensity value $\mu =\hat{\varepsilon }$ for the pME. The critical threshold *μ* from a numerical point of view arises when we find positive values of the perturbation matrix $\Delta {L}_{\varepsilon }$ that start to stress the eigenvectors orthogonal to the unperturbed stationary distribution. In particular the driving factor is the increase of terms divided by the smallest non-zero eigenvalue ${\lambda }_{F}$ of matrix *L*, that eventually causes the diverging behavior of the pME in correspondence of the critical threshold *μ*.

In order to illustrate such macroscopic change, we consider a synthetic and target network; the query nodes are randomly chosen in the synthetic setting (Fig. 2). We focus on the probability information currents *J* remaining in the network at stationary state. When an external node perturbation is introduced, the extended system reaches the perturbed stationary state ${\overrightarrow{p}}_{s}^{\ast }$ characterized by steady probability currents flowing between network nodes according to the specific features of the perturbation. We define the stationary current ${J}_{ik}$ from node *k* to node *i* as ${J}_{ik}={\pi }_{ik}{({\overrightarrow{p}}_{s}^{\ast })}_{k}-{\pi }_{ki}{({\overrightarrow{p}}_{s}^{\ast })}_{i}$. Non linearities in the stationary currents are documented by the presence of peaks of currents intensity ($I=\sum _{i,k}\,|{J}_{ik}|$) occurring at critical input values (Fig. 2c); in particular the first peak of *I* corresponds to the critical threshold *μ*. When the perturbation is small the directions of such probability currents reflect the directions found for the non-perturbed system and the currents intensity vary only by a small amount (Fig. 2d). As the input value *ε* increases toward the critical *μ*, the currents intensity grow dramatically, as the network cannot handle the exchange of information with the environment (Fig. 2e). Once *ε* passes the critical threshold the steady flow currents show a directional shift (Fig. 2f). The steady flow currents are therefore subject to a substantial change across the critical perturbation intensity *μ* confirming such value as a macroscopic turning point of the pME dynamics.

### Network frailness definition

When one sets up a pME analysis applied to real data it is convenient to set $\varepsilon$ as a parameter in order to obtain comparable results on different query node distributions and have computational advantages in the analysis of large datasets. We consider the left-hand side of Eq. (9) in order to define network frailness

10 $$\rho =\parallel \Delta {L}_{\varepsilon }\text{'}\parallel /{\lambda }_{F}$$

For each fixed source intensity $\varepsilon$ (in this work we choose $\varepsilon =1$ for simplicity), there are distributions of query nodes that may be much more disruptive than others: weak perturbations (small $\rho$) correspond to query nodes distributions associated with high network resilience (low frailness), while strong perturbations (big $\rho$) correspond to query nodes distributions associated with low network resilience (high frailness). In Eq. (10) dividing by the Fiedler number is significant because $\varepsilon ={\lambda }_{F}$ is the smallest intensity value allowing at least one query nodes distribution with diverging behavior. On the other hand in the pME model any perturbation with intensity parameter smaller than ${\lambda }_{F}$ will have a converging behavior.

### Parameters setting and data description

In the application to BC data, the coefficients ${\pi }_{kj}$ for $k,j > 0$ were determined by the stochastic transition matrix inherited by the network structure by column-normalizing the adjacency matrix of the target network; the perturbation coefficients of matrix $\Delta L$ were defined by the molecular alterations: only those nodes corresponding to altered molecular information have non-null transition rates with the environment. The exact value of such coefficients was computed by imposing $\varepsilon =1$ and assuming that all non-trivial transitions are equal (${\pi }_{k0}=1/Q$, where *Q* is the total number of query nodes); in such fashion the stochastic nature of the associated transition matrix was preserved. Finally, in matrix $\Delta L$ we chose ${\pi }_{0k}:={\pi }_{k0}$ and add ${\pi }_{0k}$ with opposite sign on the diagonal in order to keep Laplacian both the perturbation matrix ($\Delta L$) and the matrix associated to the perturbed system ($L+\Delta L$). For the expanded representation of perturbation matrix $\Delta L$ see Supplementary Information.

BC somatic mutations (SM) (Illumina Genome Analyzer platform) data were downloaded from TCGA GDC portal^38^. Mutations for a total of 926 subjects were considered in BC after outliers removal. This dataset consists of a genes-by-samples matrix ${a}_{ij}$, where, as previously done by us^2^ and others^5^, ${a}_{ij}=1$ if patient *j* has one or more mutations in gene *i* and ${a}_{ij}=0$ otherwise.

Protein-protein interactions were collected from Huri^40^. Gene identifiers were harmonized to the NCBI release. A total of 27552 interactions among 8059 genes were considered. Gene-pathway associations were downloaded from KEGG Pathway^39^. After integration of pathway information with PPI and excluding cancer pathways, a number of 92 pathways resulted containing at least a connected gene network involving at least 10 and at most 500 genes (see Supplementary Information).

### Gene frailness index (GFI)

In order to facilitate the interpretation of the applied results and provide a gene-specific index summarizing the pME results, we defined the gene frailness index (GFI). For each gene *g* of a significant gene network *W*, we quantified the co-responsibility in generating frail gene network measures by considering together the fraction of times in which *g* co-occurred mutated together with other genes in the same subject (${\sigma }_{W,g}$) and the frailness measures of patients having gene *g* mutated (${\rho }_{W,g}$). Considering now the median value ${\tilde{\rho }}_{W,g}$ among all samples presenting *g* mutated in gene network *W* we define the gene frailness index (GFI)

11 $$GF{I}_{W,g}=({\sigma }_{W,g},\frac{{\tilde{\rho }}_{W,g}}{{{\rm{\max }}}_{i}{\tilde{\rho }}_{W,i}})=({\sigma }_{W,g},{\rho {\prime} }_{W,g})$$

where we normalized the frailness component of the GFI index by the maximum ${\tilde{\rho }}_{W,g}$ among the genes of gene network *W* in order to make the index comparable also on different gene networks. The GFI can be defined also in other application fields using Eq. (11).

## Results

### Numerical properties of network frailness

We assessed the numerical properties of the introduced frailness measure (13) by considereing both synthetic and real gene networks; The query nodes were randomly chosen in the synthetic setting, while TGCA breast cancer somatic mutations were used as query nodes for the target gene network (see Methods). In particular we focused on the Apoptosis gene network because of its average size combined with the high number of BC samples presenting molecular alterations on its genes.

First, it is important to observe that a single node alteration exchanging information with the environment always registers null frailness. In fact, in the pME model it is necessary to have at least two query nodes in order to macroscopically alter the information flow on the target sub-network. This observation is a direct consequence of the assumption of the pME model and emphasizes that the method described in this work is structurally adapt to examine co-occurring molecular alterations.

**Figure 3:**
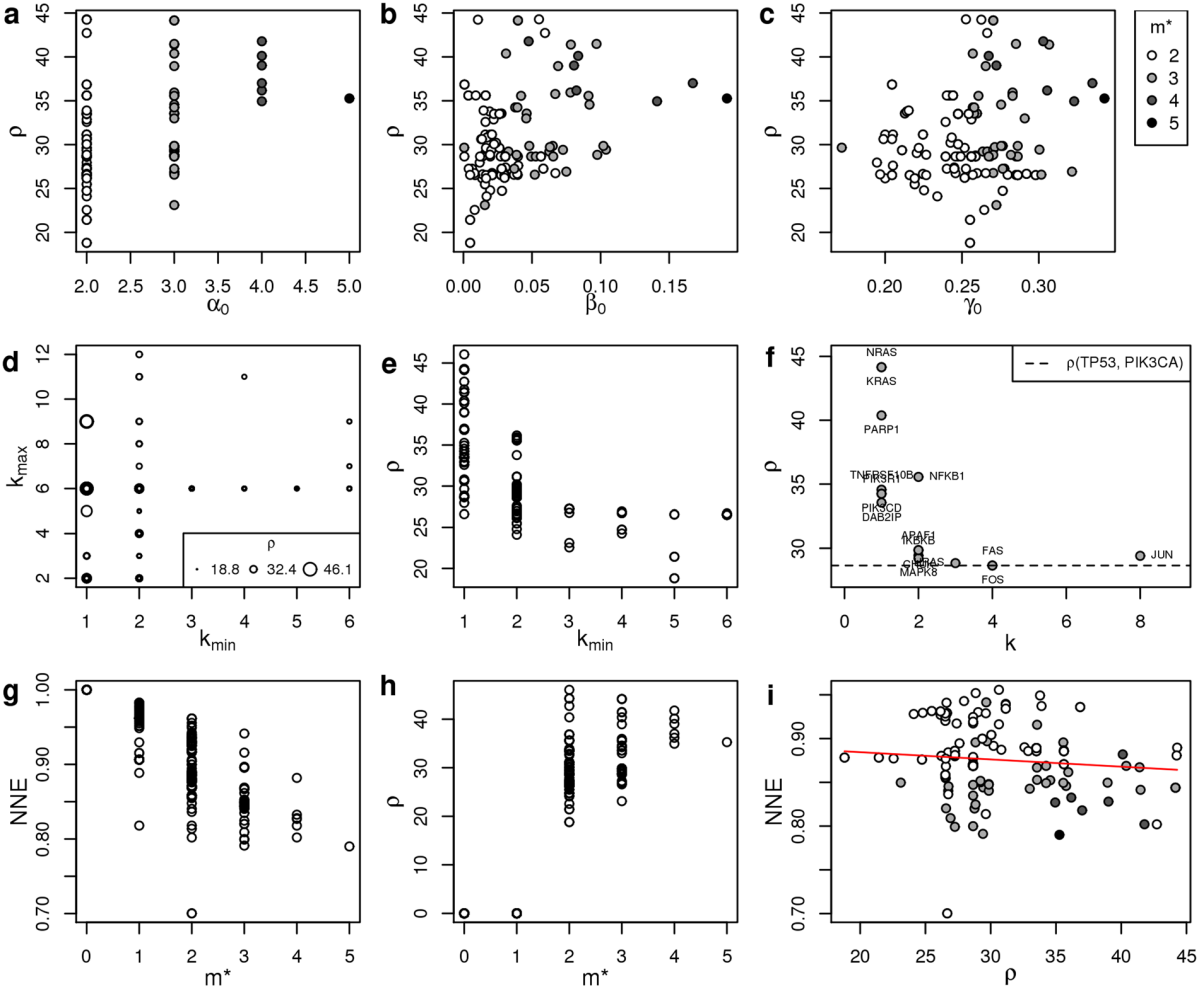
Network frailness and centrality measures. (**a–c**) Scatterplots of network frailness vs the centrality measure of the environment node 0. The centrality measures are respectively: ${\alpha }_{0}$ degree centrality, ${\beta }_{0}$ betweenness centrality, ${\gamma }_{0}$ closeness centrality. (**d**) The frailness $\rho$ plotted on corresponding minimum vs maximum node degree of each patient SM. (**e**) For each patient we plot the frailness $\rho$ vs the minimum node degree among mutated genes. (**f**) $\rho$ vs gene degree of mutated genes co-occurring with mutations in TP53 and PIK3CA in SM configurations of 3 mutated genes in real data; the horizontal dashed line corresponds to the network frailness of patients having only TP53 and PIK3CA contemporary mutated. (**g**) Normalized Network Effectivenss NNE vs number of co-mutations (${m}^{\ast }$). (**h**) Network frailness ($\rho$) vs number of co-mutations (${m}^{\ast }$). (**i**) Scatter plot of NNE vs $\rho$ on the BC samples presenting at least 2 mutations on the gene network. The solid red line represents the linear model fit. All quantities were retrieved on the Apoptosis gene network considering BC samples.

**Figure 4:**
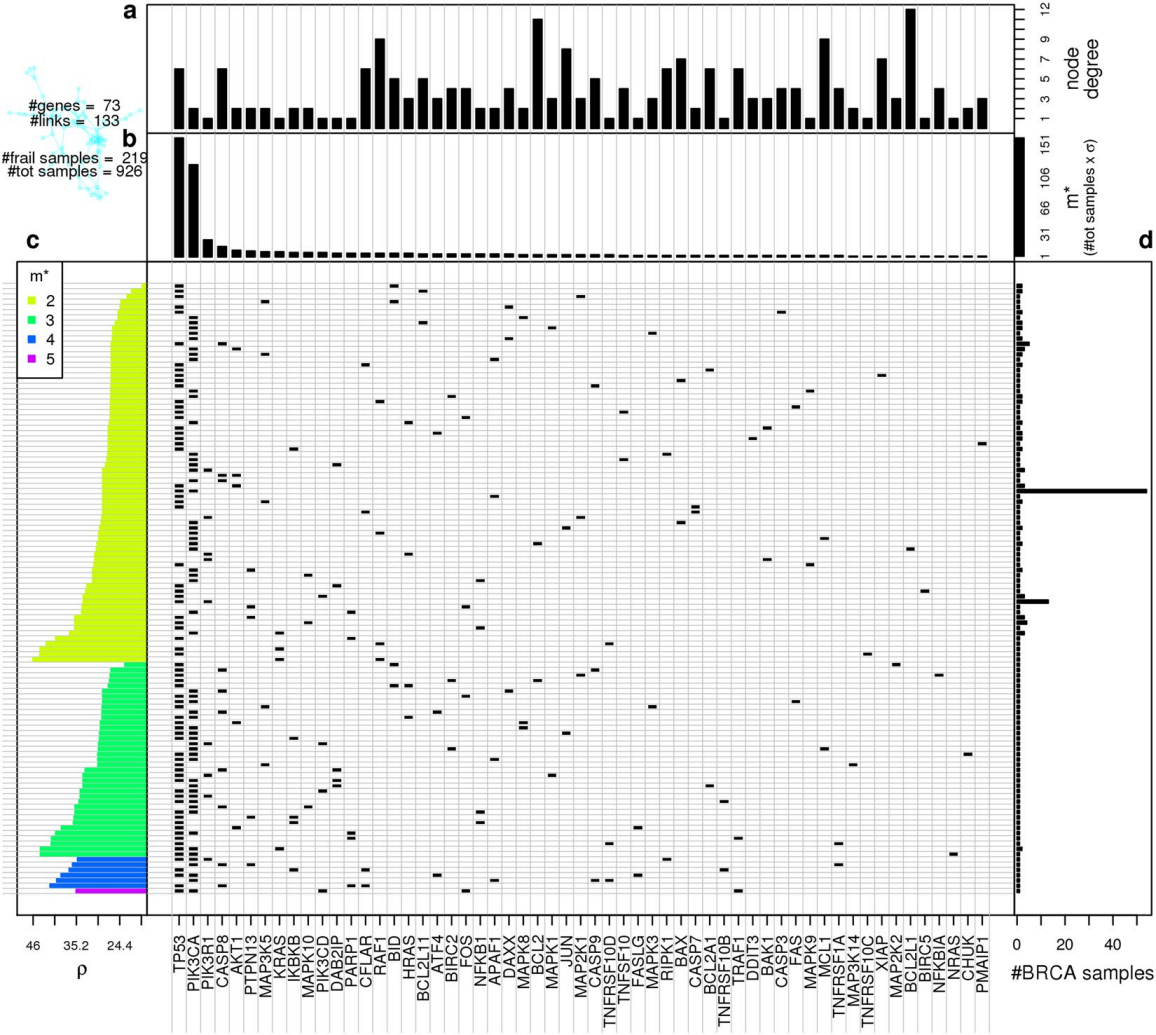
Gene network frailness analysis. Central plot: in each row we plot the somatic mutations co-occurring in the same samples on the Apoptosis gene network. Rows are ordered as the left-hand side plot, columns are sorted from left to right for decreasing ${m}^{\ast }$. Top panels: from top to bottom is respectively displayed each gene node degree in the target network (**a**) and the number of patients in which each gene is mutated together with at least another gene (${m}^{\ast }$) (**b**) the columns of the central plot are sorted according to decreasing ${m}^{\ast }$. All results were obtained using BC data mapped on KEGG Apoptosis gene network. Left-hand side panel: (**c**) Gene network frailness is plotted for each SM configuration; the ordering on the y-axis is determined in first instance by number of mutations per patient occurring on the gene network, and then in each class the configurations are sorted by $\rho$. Right-hand side panel (**d**) for each row of the central plot is displayed the number of patients presenting the corresponding SM configuration.

The second remark regards the relationship between network frailness and the number of node alterations. Indeed it is possible to identify a *monotonic property* of frailness: once fixed a connected network, there exist a straightforward dependence between the frailness *ρ* and the number of query nodes can be stated as follows:

12 $$\rho ({q}_{1},{q}_{2})\le \rho ({q}_{1},{q}_{2},{q}_{3})$$

where ${q}_{1},{q}_{2},{q}_{3}$ are different query nodes. Equation (12) is a monotonic property of *ρ* basically stating that adding a query node to existing query nodes never decreases frailness. For example, on Apoptosis gene network, all 3-mutations configurations involving both TP53 and PIK3CA never decreases *ρ* with respect to the TP53-PIK3CA two-mutations configuration (Fig. 3f). Such monotonic property for example does not exclude the case of a mutation ${q}_{4}$ such that $\rho ({q}_{1},{q}_{4}) > \rho ({q}_{1},{q}_{2},{q}_{3})$. Indeed BC patients with only two mutated genes in some cases measure a higher network frailness compared to patients presenting 3, 4 or 5 mutated genes on the same gene network (Fig. 4c).

Finally, it was crucial to investigate the relationship between network frailness and the topological position of the altered nodes on the network. In fact, the position of query nodes within a network is essential for determining why some configurations have a more destabilizing effect than others. Using Apoptosis gene network as target and BC data, we noticed non-trivial relations between the proposed measure *ρ* and classical centrality measures of the environment node: when considering the gene network extended by the new connections with the environment node (0), we observed that its degree centrality (${\alpha }_{0}$), betweenness centrality (${\beta }_{0}$), and closeness centrality (${\gamma }_{0}$) failed to predict frailness (Fig. 3a–c). This fact on one hand indicates that a wide spectrum of different topological positions of the query nodes is responsible of frail configurations; on the other hand the failure of classical topological measures in predicting *ρ* underlines the specific contribution of the pME approach.

When we considered the topological features of each query node separately, we observed that the most fragile configurations (high *ρ*) are associated with mutation profiles in which at least one query node occupies a topologically marginal position, co-occurring with more central query nodes (Fig. 3d). For example, being k the node degree, in the Apoptosis gene network the method classifies as some of the most frail mutation profiles those with mutations in TP53 or PI3KCA (k > 1) combined with mutations in genes like NRAS, KRAS and PARP1, which establish just one link within the gene network (k = 1), while for example TP53 and PIK3CA co-mutated together with FOS or FAS (k = 4) do not increase *ρ*. Therefore, considering the sets of co-mutated genes as query nodes, we can affirm that in the Apoptosis gene network the SM configurations associated with network frailness involve combinations of central genes and low-degree genes (Fig. 3d–f). This relationship between co-occurring marginal mutations and low resilience is valid also for all gene networks analyzed and discussed further.

### Network frailness and network efficiency

A measure similar to network frailness is the network efficiency (NE) of a gene network, a classical topology-based measure^42^, quantifying the impact of a set of molecular abnormalities on a target network. NE is defined as the sum of the inverse lengths of the shortest path between all network nodes divided by all the possible connections between the network nodes. Considering a set of molecular alterations we computed NE′ as the same quantity as NE calculated after removing the query nodes; since we used undirected gene networks we properly adapted the NE definition^42^. Instead of using the comparison between NE and NE′ to study the effect of multi-targets approach on pathway cross talk as in the work of Jaeger *et al*.^43^, we use the normalized network efficiency NNE = NE′/NE of a gene network hit by alterations and compare it to the proposed frailness measure *ρ*.

We first observed that while network efficiency decreases for increasing number of molecular alterations (m*) (Fig. 3g), network frailness increases (Fig. 3h), confirming that network frailness captures an increasing lack of resilience. The main difference regards samples with a single mutation: introducing a single mutation decreases network efficiency (Fig. 3g), while a single mutation does not cause any macroscopic change in the pME model therefore not increasing *ρ*. Another difference regards the jumps between distributions of measures grouped by SM numbers: when considering NNE we see a linear decrease as m* increases, while when considering *ρ* the increase is non-linear, showing that including the Laplacian properties of the network when evaluating a network disfunction leads to results that are sensibly different from classical topological approaches. In other words, the spectral properties captured by the proposed method are not easy to retrieve with classical network measures. In fact the two measures retrieved on Apoptosis pathway show an overall weak correlation (Fig. 3i), showing a sensible conceptual difference between the two measures.

### Frailness of gene networks hit by somatic mutations

The pME was applied to 92 human pathways and frailness measures were computed on all available gene networks for 926 BC patients (see Supplementary Table ST1); after mapping the patient-specific somatic mutations of BC patients on the gene networks, on 81 of them were found to have at least one BC patient with two co-occurring mutations, therefore registering non-null frailness. On average each gene network registered non-null frailness measures for approximately 57 patients (out of 926) ranging from small gene networks including single patients to big ones such as PI3K-Akt signaling pathway that included more than 400 patients. On average we found that 7 gene networks per patient to be informative about frailness, ranging from a single gene network for patients having a low number of somatic mutations to 38 gene networks for patients with many somatic mutations.

**Figure 5:**
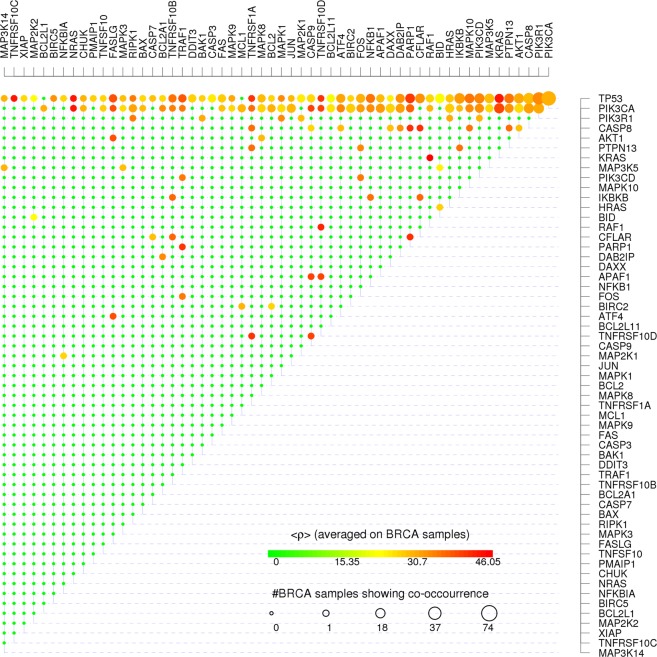
Gene-gene co-occurrences and frailness measures. For each couple of Apoptosis co-mutated genes we show the average frailness $< \rho  >$ measured on all the BC pathway having the correspondent couple of genes simultaneously mutated using the color scale from green corresponding to null frailness (maximum resilience) to red corresponding to the maximum frailness (minimum resilience) found in real BC data. The size of each dot is proportional to the log-log scaled number of BC patients presenting the co-mutation.

The results presented in Fig. 4 concerning the Apoptosis pathway show the complexity of the problem addressed. We first noticed that patients measuring high frailness are not necessarily characterized by a high number of mutated genes. Even if the average *ρ* of patients tends to increase with increasing number of SM, we found patients with only two mutated genes that cause a higher network frailness compared to patients presenting 3, 4 or 5 mutated genes (Fig. 5, left-hand panel). This fact highlights how the distribution of mutated genes more than their number determines critical issues for the information flow within the gene network. Indeed such observations show that the proposed frailness measure does not follow the *enrichment paradigm*. In order to quantify such behavior on all available gene networks we simply counted the number of 2-mutations configurations being in the most frail configurations registered. In 52% of those (56) gene networks where we registered patients with 3 or more mutations, the most frail configuration is characterized by 2 mutations only, and on all gene networks considered we found at least one 2-mutations patient with frailness higher than patients having 3 or more mutations.

Frailness is not linearly related with mutation occurrence; for instance in Apoptosis network the highest frailness across subjects is associated with the joint mutation of KRAS and RAF1, found in only one subject, while the most recurrent combination of mutations, TP53 and PIK3CA (54 patients out of the 926 considered), determines a medium frailness on this network.

An interesting issue, given a target gene network and a set of SM profiles, regards which are the most dangerous gene-gene co-mutations from suggested by the pME, also considering the restricted number of co-mutations compared to the number of all possible co-mutations (Fig. 5). For example on the Apoptosis gene network only 56 out of 73 genes are mutated in at least one patient together with another gene (the matrix showed in Fig. 5 accounts for such 56 genes only), and out of all possible gene-gene co-mutations (2628) only 116 of them (≈4%) hit the considered network.

The long-tail distribution of SM plays a major role in explaining the results showed in (Fig. 5): more than 80% of all registered SM configurations involve TP53 or PIK3CA or both (two of the most mutated genes in BC patients); on one hand we see the major role played by highly mutated genes in causing frail gene networks, on the other hand we register both a *ρ* variability within SM configurations involving highly mutated genes: the co-mutations with more rarely mutated (and most of the times marginal) genes characterizing frail SM configurations.

Indeed, the relationship between co-occurring marginal mutations and low resilience was observed for all gene networks analyzed. In order to assess statistical significance, for each gene network we considered the BC patients presenting co-occurring mutations, ranked by decreasing resilience (*ρ*). We considered only the 42 gene networks bearing signal for al least 20 patients. In the top 10 least resilient patients we found that on average 8.7 patients have at least one marginal mutation (k = 1). Using the hypergeometric test to assess significance, we found that 41 over 42 gene networks show statistical significance (p < 0.05); the only pathway not statistically significant is “Signaling pathways regulating pluripotency of stem cells” showing a p-value of 0.1, which is characterized by four top 10 frail patients having a k = 2 minimum degree mutation. More details are available in Supplementary Table ST2.

**Figure 6:**
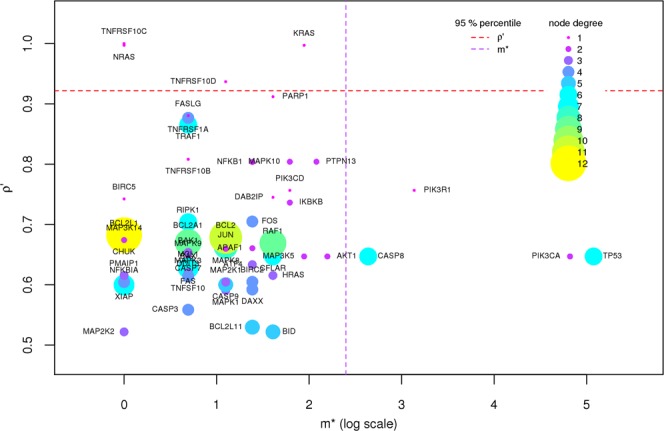
Components of the GFI. On the Apoptosis gene network we plot the GFI components for each gene of the network that has at least one non-null component. The x-axis is an elementary transformation of the gene co-occurrence $\sigma$ ($m\ast =\sigma \cdot {n}_{samples}$) and it is displayed in log scale for visualization issues. All quantities were retrieved on the Apoptosis gene network considering BC samples.

**Figure 7:**
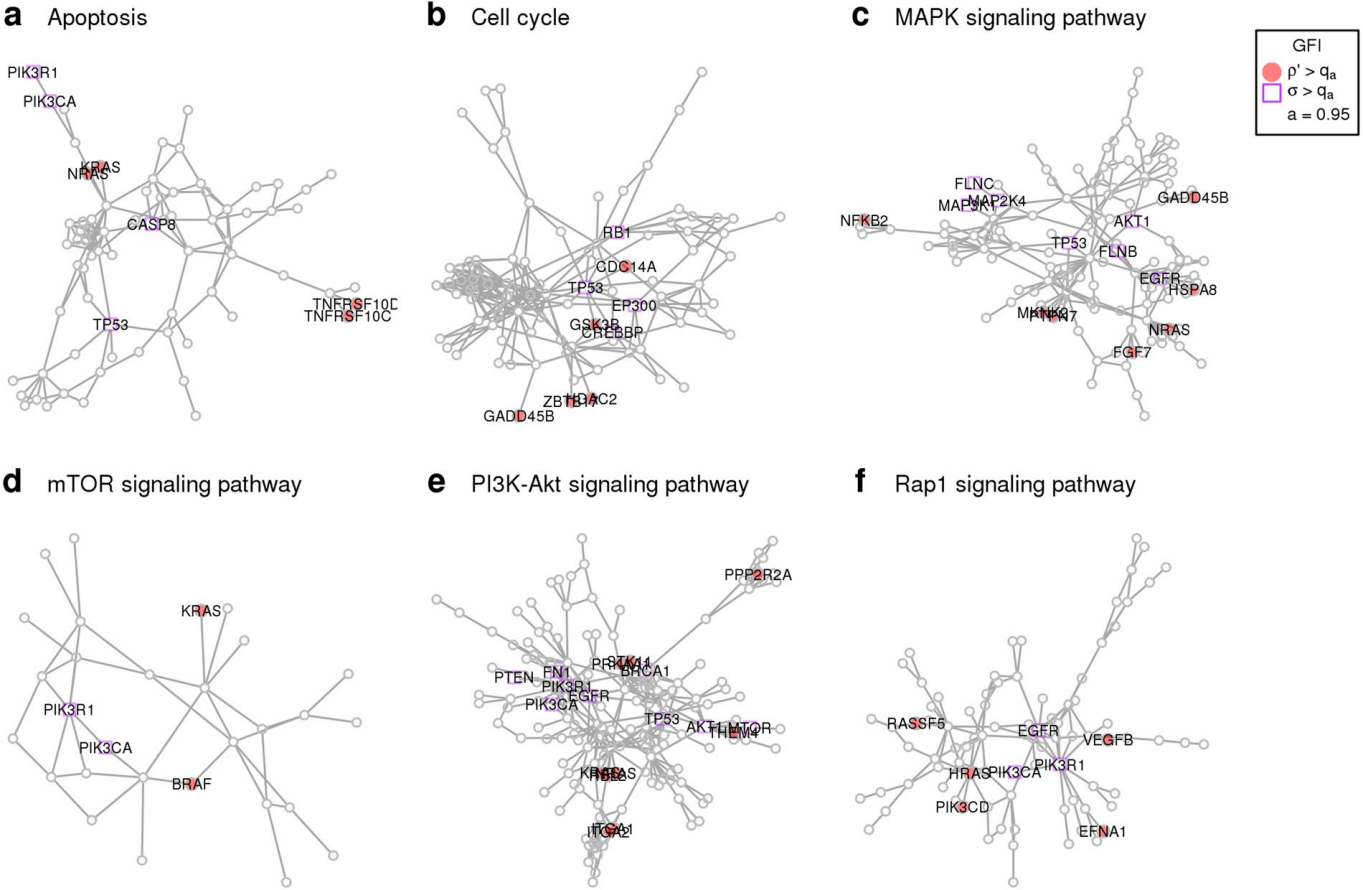
Components of GFI on KEGG gene networks. The genes presenting the highest components of GFI are are highlighted on six significant KEGG gene networks. (**a**) Apoptosis, (**b**) Cell cycle, (**c**) MAPK signaling pathway, (**d**) mTOR signaling pathway, (**e**) PI3K Akt signaling pathway, (**f**) Rap1 signaling pathay. All results are obtained using BC data.

The gene-gene co-occurrence analysis highlighted the central role of both highly mutated genes and some more rarely mutated ones in determining significant destabilizations. In order to reduce complexity and provide a gene-specific perspective, we defined GFI in the methods section (14) measuring the co-responsibility of a gene in generating frailness together with other genes. The GFI was introduced to provide a synthetic bioinformatics score summarizing the pME results. Such score was computed for all available gene networks (see Supplementary Table ST3). For the Apoptosis gene network the results of the previous analyses are highlighted by the GFI in the scatter plot (Fig. 6). Given a gene network, the GFI highlights both the genes being frequently involved in frail configurations and those genes less frequently involved in frail configurations but highly responsible of vulnerable configurations. Representative of the first class of genes on the Apoptosis gene network are TP53, PIK3CA, PIK3R1 and CASP8, while representative of the second class are NRAS, KRAS, TNFRSF10C, TNFRSF10D (Fig. 6). In fact, looking into the co-occurrences (Fig. 3) we noticed that genes such as NRAS, KRAS, TNFRSF10C, TNFRSF10D present dramatically frail measures on a few patients, while TP53, PIK3CA, PIK3R1 and CASP8 occur in many more patients presenting a high spectrum of frailness measures depending on the genes they co-occur with. Interestingly, the genes highlighted by the second component of the GFI tend to be marginal (Fig. 7a) in line with the more detailed and gene-specific analysis previously presented, and supporting the introduction of the GFI as a valid index summarizing the pME analysis.

The further detailed analysis of six selected gene networks (Apoptosis already discussed in detail, Cell cycle, MAPK signaling pathway, mTOR signaling pathway, PI3K-Akt signaling pathway and Rap1 signaling pathway) shows the applicability of the proposed method to both small networks as the mTor signaling pathway (29 genes, Fig. 7d) and bigger networks as the PI3K-Akt signaling pathway (163 genes, Fig. 7e). The genes presenting significantly high *ρ*′ component of the GFI score (red nodes in the graphs in Fig. 7) are the most interesting genes highlighted by our analysis since they are the most likely to cause network frailties in BC patients on each specific gene network. The results confirm the importance of marginal query nodes in determining high *ρ* on the gene networks and the ability of *ρ*′ to capture such behavior. Nevertheless there are also exceptions such as the central gene HRAS in Rap1 signaling pathway (Fig. 7f) being classified as highly destabilizing. The genes highlighted with the squared shape in Fig. 7 are the genes mostly mutated together with other genes in the same sample and play a major role as well as the highly destabilizing genes in determining frail gene network configurations. These considerations show how the two components of the GFI are co-essential to synthetically examine the results of the perturbative approach. For the detailed analysis of the gene networks mentioned (Cell cycle, MAPK signaling pathway, mTOR signaling pathway, PI3K-Akt signaling pathway and Rap1 signaling pathway) see Supplementary Information.

## Discussion

In this paper we introduced a novel way to study the frailness and resilience of a gene network under perturbation, based on the master equation. The method’s applicability is wide since in principle any problem that can be formulated as a perturbed system involving a prior network knowledge is suitable for the perturbed master equation (pME). For instance, with ad hoc modifications, the pME framework could be adapted to model perturbations of financial or transportation networks. In this context, we presented a proof-of-principle in the system medicine field using a database of breast cancer mutations and a series of representations of pathways to illustrate the applicability to real data and to underline the complexity of the problem.

The formulation of the pME model is characterized by the assumption that network nodes exchange information according to the master equation as general and relatively simple mathematical framework. Information is therefore thought as an ideal substance that moves along network edges according to the master equation; depending on the specific application field (e.g. transportation, biochemical or financial networks) the appropriate form of the master equation may change accordingly. In order to provide a robust framework for the modeling of intra-cellular dynamics we chose to compute the Laplacian of the master equation^41^ only by considering the given network structure^40^. We therefore strongly relied on the physical interactions between molecular entities retrieved experimentally to define transitions among molecular entities. The main peculiarity of the pME is that, when a network is perturbed, the (given) node alterations exchange information with a node external to the system that represents the environment. In this fashion, the information flow is somehow polarized by the node alterations simultaneously exchanging information with the environment. The numerical study of the pME, highlighted the presence of a critical intensity of information exchange with the environment that captures a macroscopic change in the system.

Basing ourselves on the pME model, we defined network frailness, quantifying how much a given distribution of node alterations on a target network may alter the information exchange within the network. In this paper we showed the failure of classical centrality measures in predicting frailness, pointing out that the spectral properties captured by the proposed method are not easy to retrieve with classical network measures, as it emerges also from the comparison with network efficiency (NNE).

For the application, we considered BC data downloaded from TCGA database^38^. Focusing on each single BC patient, the mutated genes were interpreted as query nodes of a selected target network: such gene networks were obtained by the largest connected components of the subnetworks of the protein-protein interaction defined by KEGG human pathways^39^. The results were obtained on 92 different gene networks: in order to present the applicability of the proposed method we focused on the Apoptosis gene network and a few others. To examine the results from a gene perspective, we defined the gene frailness index (GFI) a measure that reflects both the gene mutation co-occurrence in disease-related samples and their co-responsibility in causing high frailness configurations. Even if an approximation of the pME, such a measure is able to capture the key results of the network frailness analysis. The definition of the GFI paves the way for a novel network-based pathway analysis. For simplicity in this work we chose to focus on the pME definition and applicability and leave the development of a comprehensive frailness-based pathway analysis to future work.

We observed a relevant variation among frailness measures determined by the mutation of the same gene across mutation profiles, according to which other genes are co-mutated. We noticed that SM profiles with mutations in central genes only have a less disruptive impact on resilience compared to mutation profiles with a combination of mutations in central and low degree genes. In particular we can affirm that a mutation profile with at least one mutation in a low-degree gene (independently from the centrality of the other co-mutating genes) have the highest ability to drive the system far from the expected stationary state. Interestingly, also related literature underlines the role of marginal nodes in the control range context^36^; the importance of marginal nodes was also recently addressed regarding the control of multilayer scale-free networks^44^. In biological networks like the ones considered in this work, low-degree genes can represent, depending on how the network is defined, relevant pathway regulators, as growth factors. The combinations of mutated genes marked as less resilient could suggest possible drug targets for controlling the activity of the gene network.

In general, the method presented in this work can be experimentally validated by comparing the activity of the real system with that of the same system after perturbation. Clearly, the suitability of the gene network used to represent the real system would constitute a crucial factor to obtain useful predictions from the method presented in this work. In the proof-of-principle, we studied Apoptosis and other pathways using generic representations provided by KEGG^39^ and protein-protein interactions^40^. However, in a future study focused on the frailness of a process like Apoptosis, a more tailored gene network can be considered adding mechanistic details that would better model the real process. In that case, the frailness of Apoptosis, due to a series of different combinations of mutated genes, can be assessed in an *in vitro* cellular model, comparing the apoptotic process of cells with altered activity (e.g. over expression) of mutated genes with apoptosis of the same cell type without mutations.

The proposed method, even if with important formal differences, presents in principle a strong link to classical control theory because the pME represents a framework where it is reasonable to investigate which distributions of query nodes lead to a macroscopic change of state and which not. Such approach would allow to characterize the frail query nodes combinations as the ones easily leading the network to a macroscopic change of state. In terms of biological applications these concepts translate for example to the definition of the sets of molecular alterations that lead to a final state that is critical for the network information flow, therefore likely to be associated with a given disease. The formal definition, the development, and the applications of a perturbation-based network control model is left to future work.

## Conclusion

In this work we defined a new approach to the quantification of frailness and resilience of complex networks based on a perturbed master equation. The application of the pME to both synthetic and real data, showed that frailness depends on the position of the alterations on the network more than on their amount, unlike most traditional enrichment scores. In particular low-degree alterations play an important role in causing high frailness measures. In this perspective, considering breast cancer samples, we observed the key role of marginal alterations such as as growth factors in determining frailness, together with other well known frequently mutated genes. The potential applicability of the proposed method is wide and suggests future development in the control theory context.

## Supplementary information

Supplementary Information.

Supplementary Table ST1.

Supplementary Table ST3.
