# Supplementary material for: Frailness and resilience of gene networks predicted by detection of co-occurring mutations via a stochastic perturbative approach
Source: Sci Rep. 2020 Feb 14;10:2643. doi: 10.1038/s41598-020-59036-w (PMC7021762; doi:10.1038/s41598-020-59036-w)
Supplement: Supplementary file 1 — Supplementary Information. [file 41598_2020_59036_MOESM1_ESM.docx]

**Frailness and resilience of gene networks predicted by detection of co-occurring mutations via a stochastic perturbative approach**

Matteo Bersanelli^1,2,*^, Ettore Mosca^3^, Luciano Milanesi^3^, Armando Bazzani^1^, and Gastone Castellani^1^

^1^Department of Physics and Astronomy, University of Bologna, Bologna 40127, Italy

^2^National Institute for Nuclear Physics (INFN), Bologna 40127, Italy

^3^Institute of Biomedical Technologies, National Research Council, Segrate (Milan) 20090, Italy

* matteo.bersanelli2@unibo.it

**Supplementary Information**

**Supplementary Note**

**pME model derivation.** We consider the master equation on an M-nodes network where $\pi_{kj}$ is the information transition rate between node j and node k. Let $p_{k}(t)$ be the probability to find the node k is active at time t; the ME reads

$\dot{p_{k}}=\sum_{j} (\pi_{kj}p_{j}-\pi_{jk}p_{k}), k=1,\ldots,M$ (S1)

that corresponds to the continuity equation for the probability distribution with the constraint $\sum_{k} p_{k}\left( t \right)=1.$ It is convenient to introduce the vector notation $\vec{p}={(p_{1},\ldots,p_{M})}^{T}$ and to define the Laplacian matrix of the network

$L_{kj}= d_{k}\delta_{kj}-\pi_{kj}, d_{k}=\sum_{j} \pi_{jk}$ (S2)

so that the master equation reads as equation (1), and the stationary solution $\vec{p}_{s}$ corresponds to the kernel of the matrix *L*. In a generic case *L* has all positive eigenvalues except the the first one which is zero, so that the stationary state is unique and attractive. Different methods can be applied to solve equation (1) without computing the characteristic equation of the matrix L.

We now consider the presence of a network perturbation as an external node (0) playing the role of the environment from which information can be introduced into the network and to which particles can flow out from the current network. Defining the novel connections from ($\pi_{k0}$) and to ($\pi_{0k}$) the environment node we can write an extended master equation for the perturbed state in compact form as Equation 2, where $L_{0}$ is the extension of the Laplacian matrix $L$ where the environment node is decoupled from the existing network, while $\Delta L$ is the perturbation matrix defining the connections with the environment:

$$L_{0}=\left( \begin{matrix} 0 & \begin{matrix} 0 & \ldots& 0 \end{matrix} \\ \begin{matrix} 0 \\ \vdots\\ 0 \end{matrix} & L \end{matrix} \right), \Delta L=\left( \begin{matrix} \begin{matrix} \varepsilon\\ {-\pi}_{10} \end{matrix} \\ \begin{matrix} {-\pi}_{20} \\ \vdots\end{matrix} \\ \begin{matrix} \vdots\\ {-\pi}_{M0} \end{matrix} \end{matrix}\begin{matrix} \begin{matrix} -\pi_{01} \\ \pi_{01} \end{matrix} \\ \begin{matrix} 0 \\ \vdots\end{matrix} \\ \begin{matrix} \vdots\\ 0 \end{matrix} \end{matrix}\begin{matrix} \begin{matrix} -\pi_{02} \\ 0 \end{matrix} \\ \begin{matrix} \pi_{02} \\ 0 \end{matrix} \\ \begin{matrix} \vdots\\ 0 \end{matrix} \end{matrix}\begin{matrix} \begin{matrix} \ldots\\ \ldots\end{matrix} \\ \begin{matrix} \ldots\\ \ddots\end{matrix} \\ \begin{matrix} \ddots\\ \ldots\end{matrix} \end{matrix}\begin{matrix} \begin{matrix} {-\pi}_{0M-1} \\ \ldots\end{matrix} \\ \begin{matrix} \ddots\\ \ddots\end{matrix} \\ \begin{matrix} \pi_{0M-1} \\ 0 \end{matrix} \end{matrix}\begin{matrix} \begin{matrix} -\pi_{0M} \\ 0 \end{matrix} \\ \begin{matrix} \vdots\\ \vdots\end{matrix} \\ \begin{matrix} 0 \\ \pi_{0M} \end{matrix} \end{matrix} \right)$$

where $\varepsilon=\sum_{j} \pi_{j0}$ in order to set both $L_{0}$ and $\Delta L$ as M+1 x M+1 Laplacian matrices and the vector $\vec{p}={(p_{0},p_{1}, \ldots, p_{M})}^{T}$ is now M+1 dimensional as well. If we set $\varepsilon=1$, we preserve the stochastic character of the matrix $\pi_{jk}$. We omit the subscript $\varepsilon$ in matrix $\Delta L$, unlike equation (2) in the main text. Once fixed the configuration of connections to and from the environment, equation (2) has a unique stationary solution $\vec{p}_{s}^{*}$ that can be computed directly. It is interesting to compute the condition which preserves the stationary solution $\vec{p}_{s}$ of the unperturbed system (1). By a direct substitution we have

$\frac{\pi_{k0}}{\varepsilon}- \pi_{0k}p_{k}^{s}=0 \forall k$ (S3)

that is equivalent to a detailed balance condition between the source and each sink, so that the probability flow is null for each link.

**Quantifying network frailty.** We look directly at the convergence condition for equation (7) in the main manuscript. We operate a direct substitution

$c_{n,k}=-\frac{1}{\lambda_{k}}\sum_{j} \left[ \bar{\Delta L}_{kj}-\frac{\bar{\Delta L}_{k1}}{\bar{\Delta L}_{11}}\bar{\Delta L}_{1j} \right]c_{n-1,j}+b_{n,k}$ (S4)

where $b_{n,k}$ do not depend on the coefficients $c_{n-1,j}$. So we consider the matrix in the square brackets:

${\Delta L'}_{kj}:= \bar{\Delta L}_{kj}-\frac{\bar{\Delta L}_{k1}}{\bar{\Delta L}_{11}}\bar{\Delta L}_{1j}$ (S5)

the iterative scheme recurrence is driven by $\Delta L'$ and the inverse of matrix $L$ in the subspace orthogonal to its kernel so that the condition for equation (7) of the main manuscript to be a contraction becomes:

$\left\| L^{-1} \right\| \left\| \Delta L' \right\|<1 \underset{\Leftrightarrow}{} \left\| \Delta L' \right\|<$ $\lambda_{F}$ (S6)

where we are considering a classical matrix norm

$$\left\| M \right\|:=\sup_{v} \frac{|Mv|}{v}$$

when $M$ is symmetric its norm corresponds to the absolute value of its biggest eigenvalue.

The right-hand side of equation (S6) is reported in equation (9) of the main text. Thanks to such convergence condition we retrieve the network frailness using the matrix norm $\left\| \Delta L' \right\|$. We define the quantity $\rho=\left\| \Delta L' \right\|/\lambda_{F}$ in equation (10) of the main manuscript as the frailness of a network; $\lambda_{F}$ is the Fiedler number of the unperturbed network (the smallest non-zero eigenvalue of the associated Laplacian matrix).

**Gene Networks.** For the analysis we considered 92 gene networks obtained by integrating KEGG human pathway information (selected as explained in the main text) and Huri protein-protein interactions. The pathways where at least one BRCA patient has at least 2 mutated genes are 81.

Here is the complete list: Purine metabolism, Pyrimidine metabolism, Basal transcription factors, Proteasome, Base excision repair, Nucleotide excision repair, Mismatch repair, Homologous recombination, **MAPK signaling pathway**, ErbB signaling pathway, Calcium signaling pathway, Cytokine-cytokine receptor interaction, **Cell cycle**, p53 signaling pathway, Ubiquitin mediated proteolysis, Autophagy - animal, **mTOR signaling pathway**, **Apoptosis**, Wnt signaling pathway, Notch signaling pathway, TGF-beta signaling pathway, VEGF signaling pathway, Focal adhesion, Adherens junction, Tight junction, Gap junction, Complement and coagulation cascades, Toll-like receptor signaling pathway, Jak-STAT signaling pathway, Natural killer cell mediated cytotoxicity, T cell receptor signaling pathway, B cell receptor signaling pathway, Fc epsilon RI signaling pathway, Leukocyte transendothelial migration, Long-term potentiation, Long-term depression, Regulation of actin cytoskeleton, Insulin signaling pathway, GnRH signaling pathway, Progesterone-mediated oocyte maturation, Melanogenesis, Chemokine signaling pathway, Lysosome, Neurotrophin signaling pathway, Endocytosis, Fc gamma R-mediated phagocytosis, RIG-I-like receptor signaling pathway, NOD-like receptor signaling pathway, Spliceosome, Oocyte meiosis, Peroxisome, Protein processing in endoplasmic reticulum, RNA transport, mRNA surveillance pathway, Fanconi anemia pathway, Serotonergic synapse, NF-kappa B signaling pathway, **PI3K-Akt signaling pathway**, HIF-1 signaling pathway, Hippo signaling pathway, Estrogen signaling pathway, TNF signaling pathway, Prolactin signaling pathway, Ras signaling pathway, **Rap1 signaling pathway**, Adrenergic signaling in cardiomyocytes, FoxO signaling pathway, Thyroid hormone signaling pathway, Platelet activation, Oxytocin signaling pathway, AMPK signaling pathway, cAMP signaling pathway, Signaling pathways regulating pluripotency of stem cells, Sphingolipid signaling pathway, Glucagon signaling pathway, Phospholipase D signaling pathway, Apoptosis - multiple species, Th1 and Th2 cell differentiation, Th17 cell differentiation, IL-17 signaling pathway, Apelin signaling pathway.

In the prevoius list we highlighted the 6 pathways chosen for detailed analysis. The same results of all the 75 remaining gene networks are retrievable from **Supplementary Table ST1, ST3**.

**Supplementary Tables**

**Supplementary Table ST1.** The results of the pME analysis are reported on all available BC patients and all gene networks considered. Columns description (from left to right): **pathway** = name of the KEGG pathway used to identify significant gene network; **patients** = TCGA id of the patients presenting signal on correspondent pathway; **genes** = mutated genes in correspondent BC patient; **rho** and **sigma** correspond respectively to the frailness measure computed as equation (10) and $\sigma$ is the fraction of times a gene co-occurs with other genes in the correspondent gene network; **mut** = number of patients presenting the corresponding mutation on the given gene network; **degree** = node degree of mutated gene in the given gene network

**Supplementary Table ST2.** Statistical assestment of marginal mutations impact on frailness: hypergeometric test results. **TFP** = number of top frailness patients considered as target set for hypergeometric test; **PA** = number of pathways available for hypergeometric test. **M (%)** = average percentage (on PA) of profiles with at least one marginal gene (k=1) in TFP. **p < 0.2 (%)** = percentage of PA on which p-value is smaller than 0.2; **p < 0.1 (%)** = percentage of PA on which p-value is smaller than 0.1; **p < 0.05 (%)** = percentage of PA on which p-value is smaller than 0.05.

| **TFP** | **PA** | **M (%)** | **p < 0.2 (%)** | **p < 0.1 (%)** | **p < 0.05 (%)** |
| --- | --- | --- | --- | --- | --- |
| 5 | 53 | 93.2% | 98.1% | 98.1% | 96.2% |
| 10 | 42 | 94.3% | 100% | 97.6% | 97.6% |
| 15 | 40 | 91.2% | 100% | 100% | 100% |
| 20 | 32 | 88.1% | 100% | 93.8% | 93.8% |

**Supplementary Table ST3.** The results of the GFI analysis are reported on all available BC patients and all gene networks considered. Columns description (from left to right): **pathway** = name of the KEGG pathway used to identify significant gene network; **genes** = mutated genes in correspondent BC patient; **rho1** and **sigma** correspond respectively to the components $\rho'$ and $\sigma$ of the GFI as computed in equation (11); **mut** = number of patients presenting the corresponding mutation on the given gene network; **degree** = node degree of mutated gene in the given gene network

**Supplementary Figures**

**----------------------------------- Cell Cycle ----------------------------------------**

**Supplementary Figure S1**

**
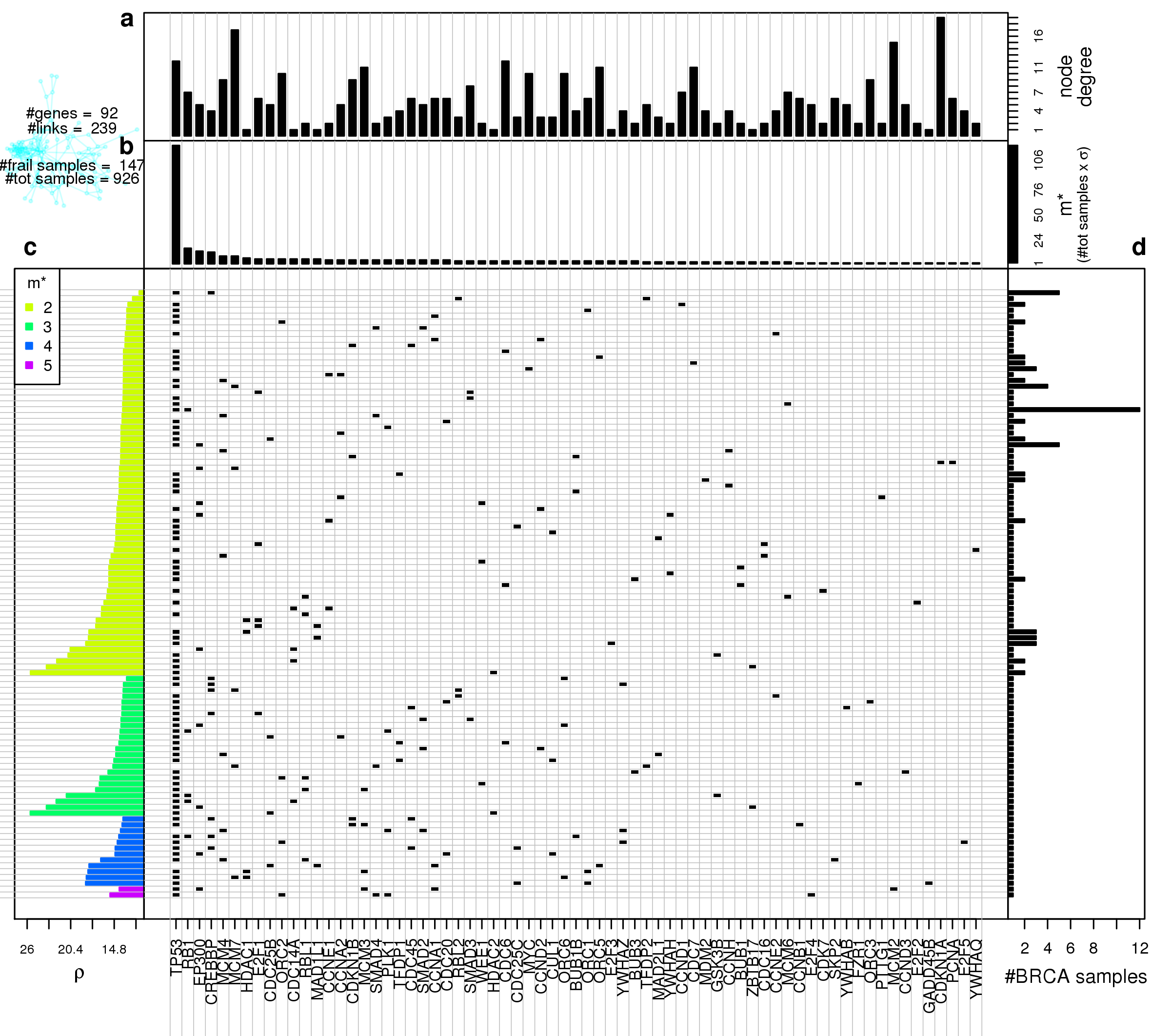
**

**Figure S1. Gene network frailness analysis for Cell Cycle.** Central plot: in each row we plot the somatic mutations co-occurring in the same samples on the Cell Cycle gene network. Rows are ordered as the left-hand side plot, columns are sorted from left to right for decreasing *m*∗. Top panels: from top to bottom is respectively displayed each gene node degree in the target network (a) and the number of patients in which each gene is mutated together with at least another gene (*m*∗) (b); the columns of the central plot are sorted according to decreasing *m*∗. All results were obtained using BC data mapped on KEGG’s Cell Cycle gene network. Left-hand side plot (c): Gene network frailty is plotted for each SM configuration; the ordering on the y-axis is determined in first instance by number of mutations per patient occurring on the gene network, and then in each class the configurations are sorted by ρ. Right-hand side plot (d): for each row of the central plot is displayed the number of patients presenting the corresponding SM configuration.**Supplementary Figure S2**

**
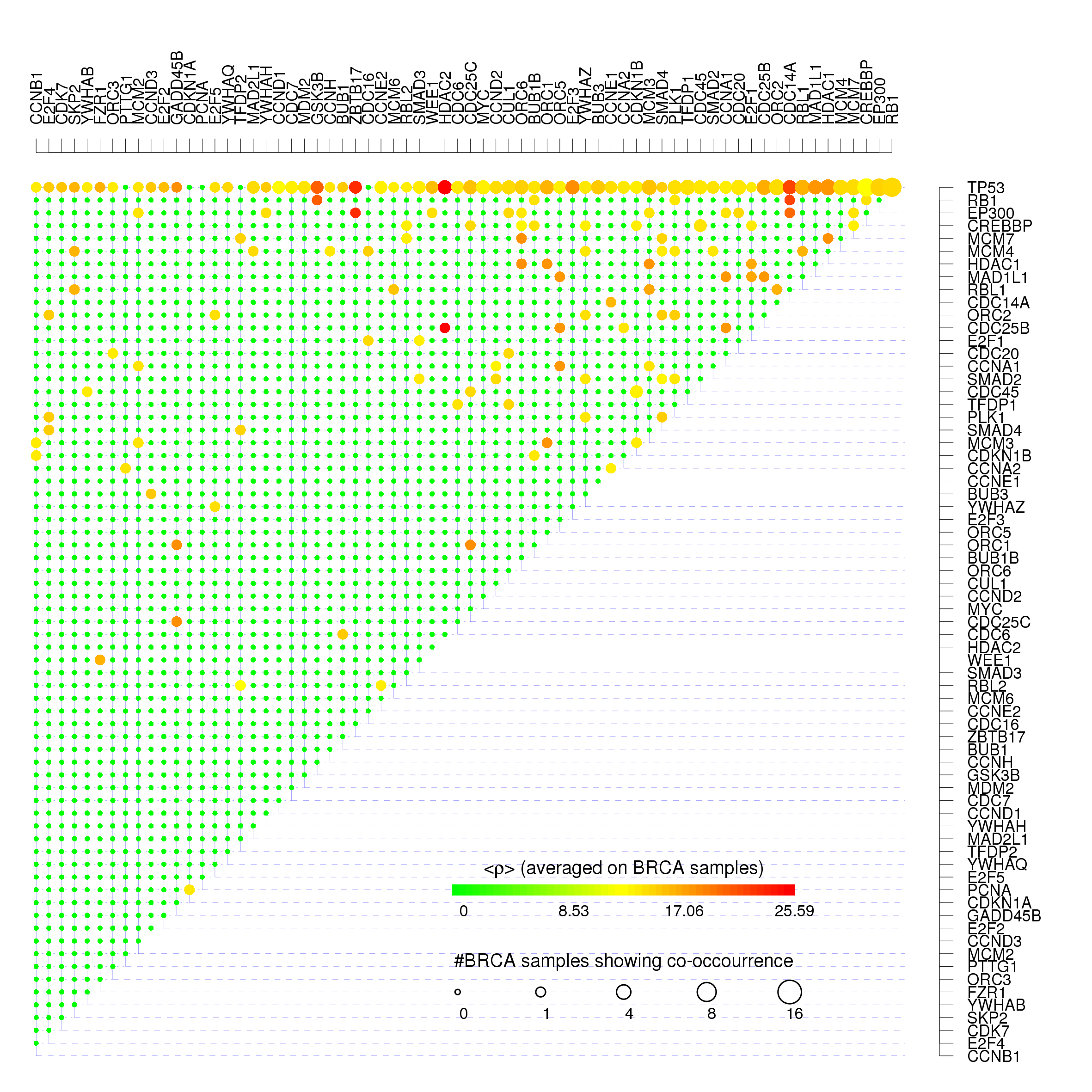
**

**Figure S2. Gene-gene co-occurrences and frailness measures for Cell Cycle.** For each couple of Cell Cycle co-mutated genes we show the average frailty < ρ > measured on all the BC pathway having the correspondent couple of genes simultaneously mutated using the color scale from green corresponding to null frailty (maximum resilience) to red corresponding to the maximum frailty found in real BC data. The size of each dot is proportional to the log-log scaled number of BC patients presenting the co-mutation.

**Supplementary Figure S3**

**
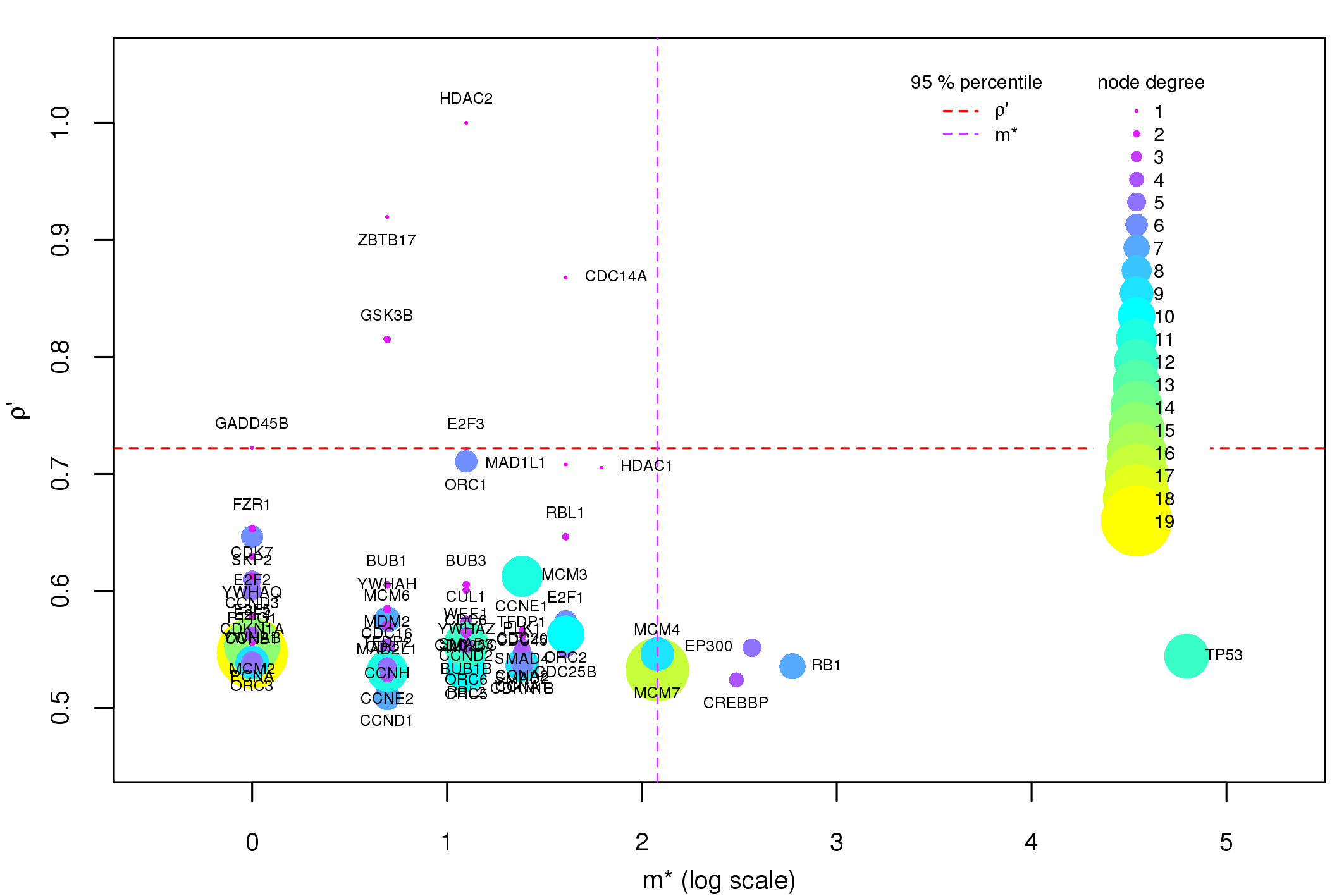
**

**Figure S3. Components of the GFI for Cell Cycle**. On the Cell Cycle gene network we plot the GFI components for each gene of the network that has at least one non-null component. The x-axis is an elementary transformation of the gene co-occurrence σ (*m*∗ = σ · *nsamples* ) and it is displayed in log scale for visualization issues.

**------------------------- MAPK signaling pathway -----------------------------**

**Supplementary Figure S4**

**
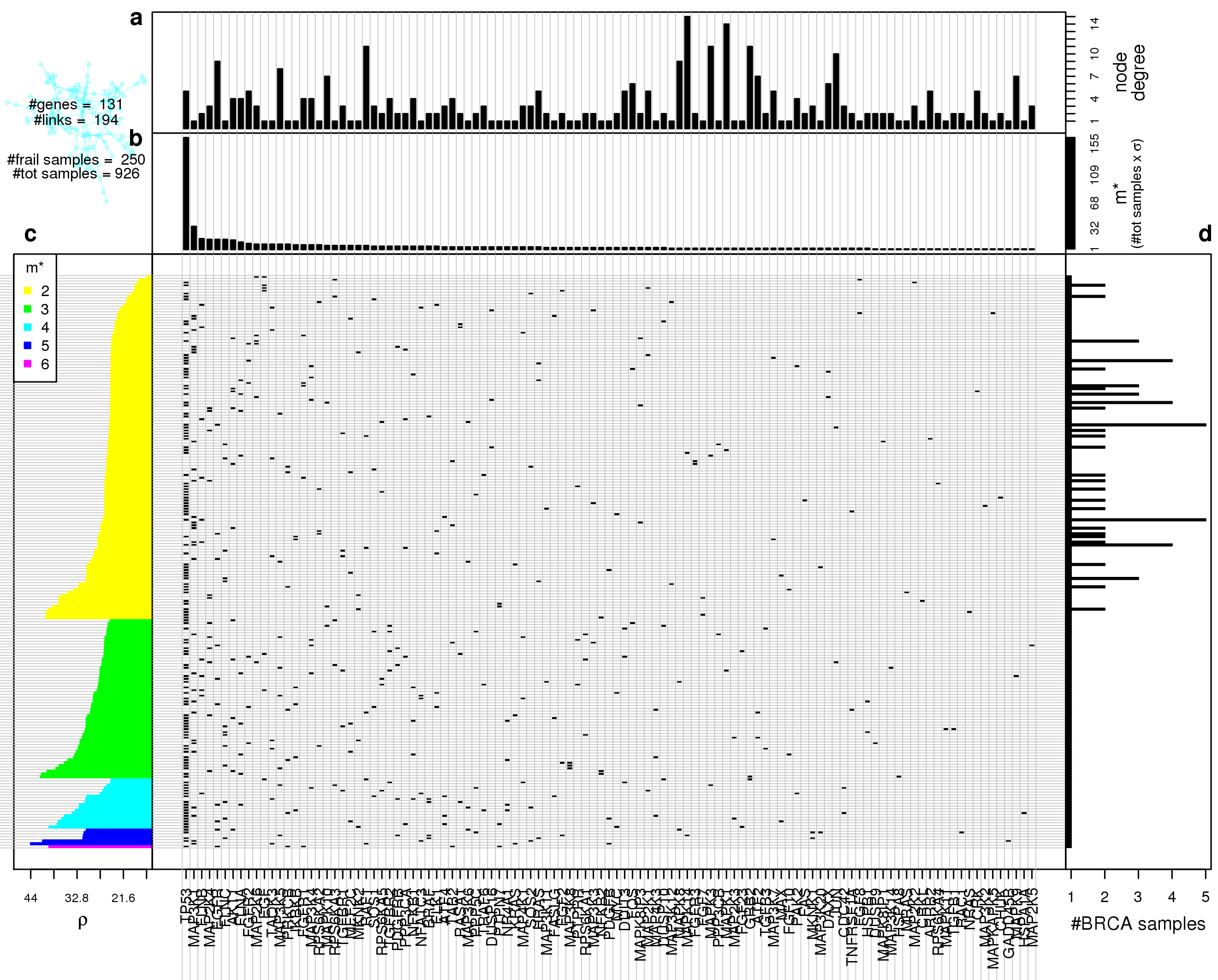
**

**Figure S4. Gene network frailty analysis for** **MAPK signaling pathway.** Central plot: in each row we plot the somatic mutations co-occurring in the same samples on the MAPK signaling pathway gene network. Rows are ordered as the left-hand side plot, columns are sorted from left to right for decreasing *m*∗. Top panels: from top to bottom is respectively displayed each gene node degree in the target network (a) and the number of patients in which each gene is mutated together with at least another gene (*m*∗) (b); the columns of the central plot are sorted according to decreasing *m*∗. All results were obtained using BC data mapped on KEGG’s MAPK signaling pathway gene network. Left-hand side plot (c): Gene network frailty is plotted for each SM configuration; the ordering on the y-axis is determined in first instance by number of mutations per patient occurring on the gene network, and then in each class the configurations are sorted by ρ. Right-hand side plot (d): for each row of the central plot is displayed the number of patients presenting the corresponding SM configuration.

**Supplementary Figure S5**

**
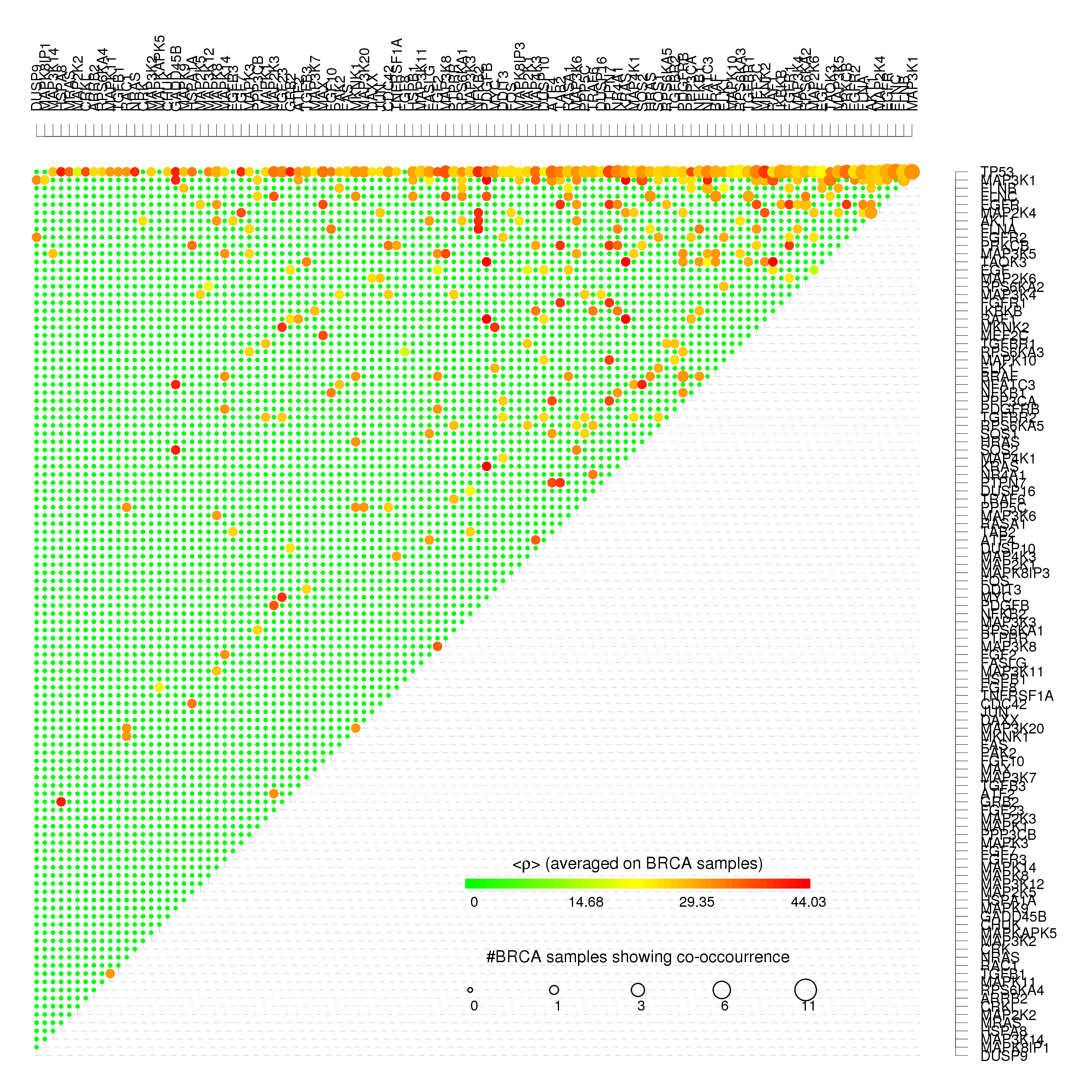
**

**Figure S5. Gene-gene co-occurrences and frailty measures for** **MAPK signaling pathway.** For each couple of MAPK signaling pathway co-mutated genes we show the average frailty < ρ > measured on all the BC pathway having the correspondent couple of genes simultaneously mutated using the color scale from green corresponding to null frailty (maximum resilience) to red corresponding to the maximum frailty found in real BC data. The size of each dot is proportional to the log-log scaled number of BC patients presenting the co-mutation.

**Supplementary Figure S6**

**
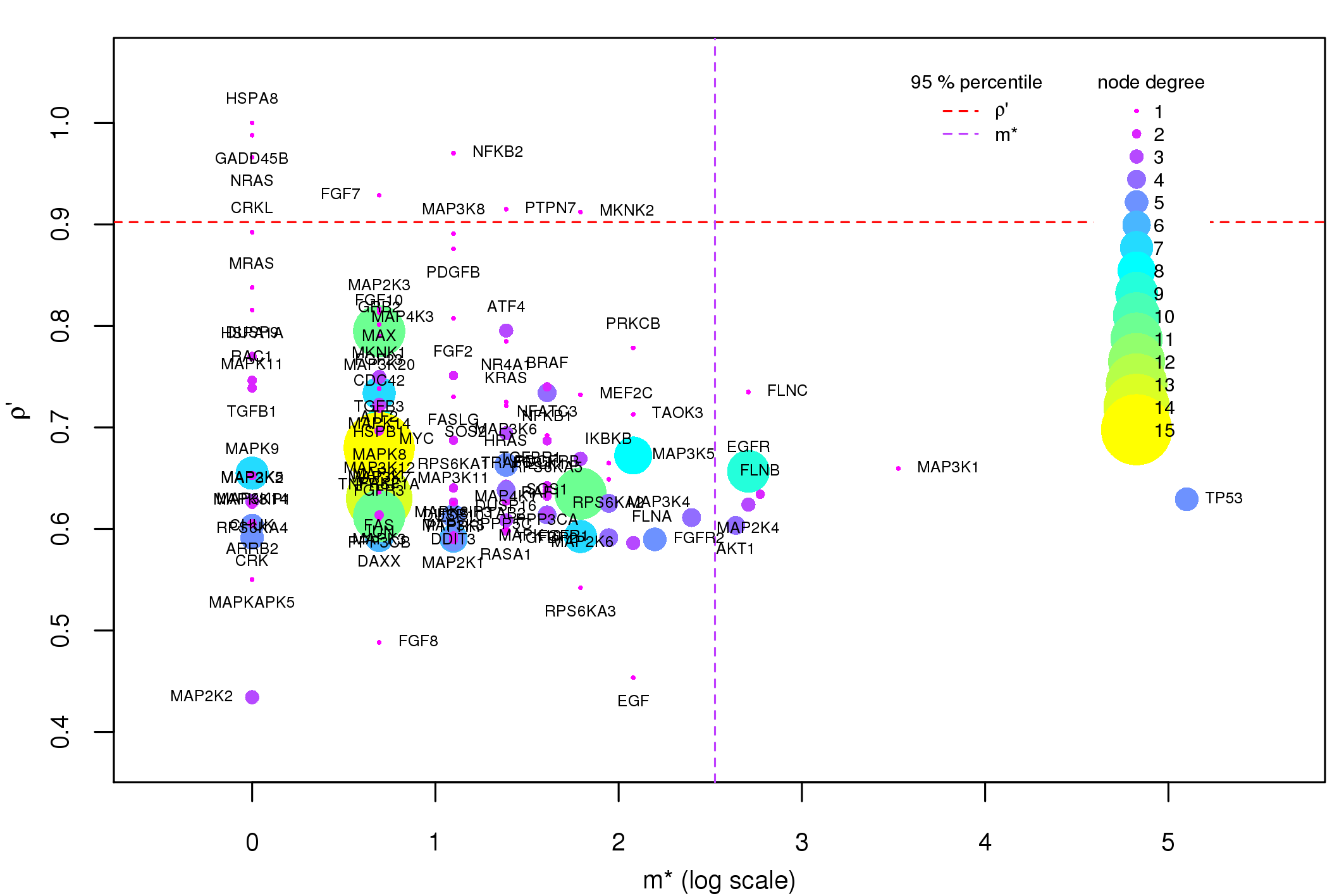
**

**Figure S6. Components of the GFI for** **MAPK signaling pathway**. On the MAPK signaling pathway gene network we plot the GFI components for each gene of the network that has at least one non-null component. The x-axis is an elementary transformation of the gene co-occurrence σ (*m*∗ = σ · *nsamples* ) and it is displayed in log scale for visualization issues.

**------------------------- mTor signaling pathway -------------------------------**

**Supplementary Figure S7**

**
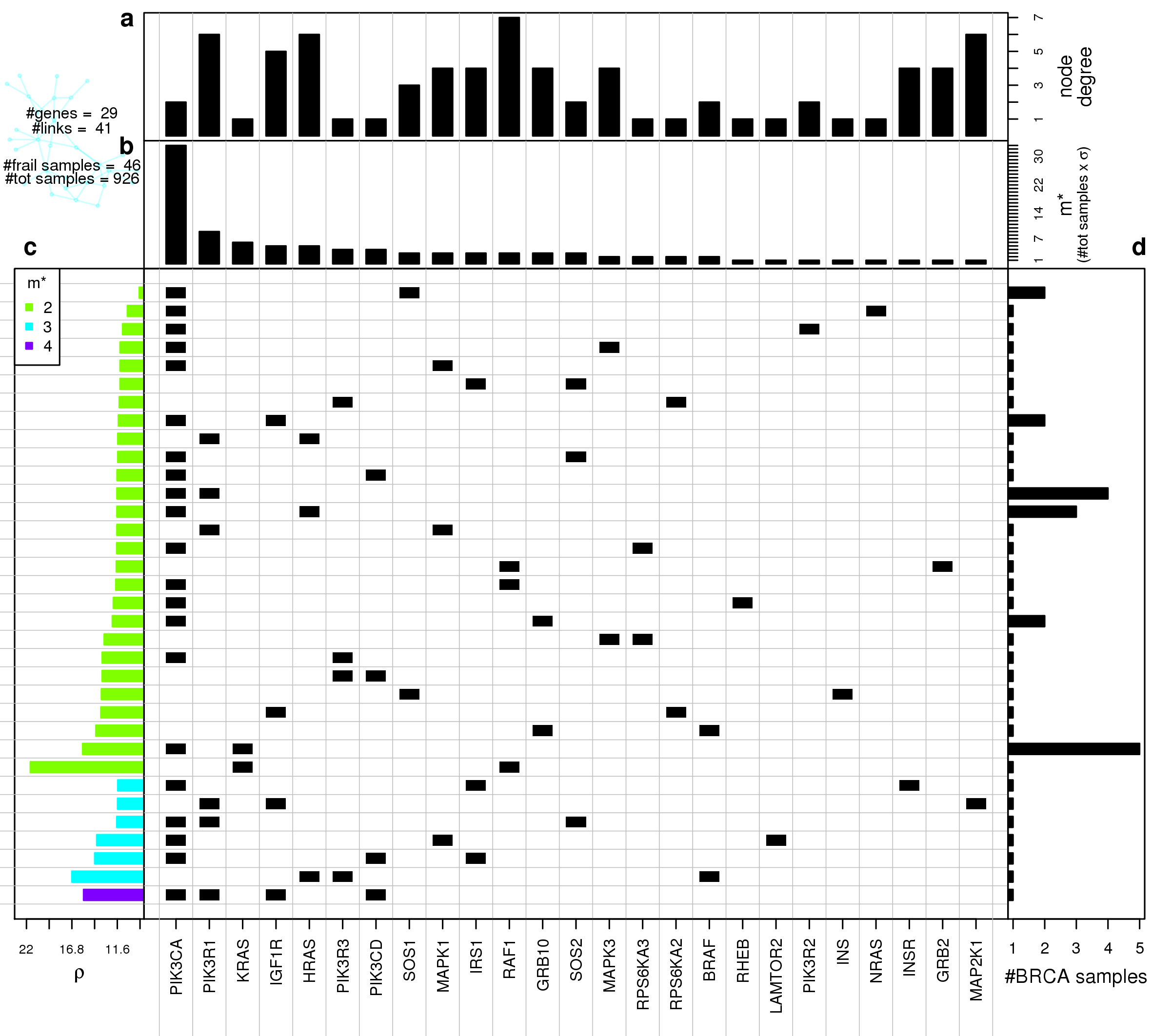
**

**Figure S7. Gene network frailty analysis for mTor signaling pathway.** Central plot: in each row we plot the somatic mutations co-occurring in the same samples on the mTor signaling pathway gene network. Rows are ordered as the left-hand side plot, columns are sorted from left to right for decreasing *m*∗. Top panels: from top to bottom is respectively displayed each gene node degree in the target network (a) and the number of patients in which each gene is mutated together with at least another gene (*m*∗) (b); the columns of the central plot are sorted according to decreasing *m*∗. All results were obtained using BC data mapped on KEGG’s mTor signaling pathway gene network. Left-hand side plot (c): Gene network frailty is plotted for each SM configuration; the ordering on the y-axis is determined in first instance by number of mutations per patient occurring on the gene network, and then in each class the configurations are sorted by ρ. Right-hand side plot (d): for each row of the central plot is displayed the number of patients presenting the corresponding SM configuration.

**Supplementary Figure S8**

**
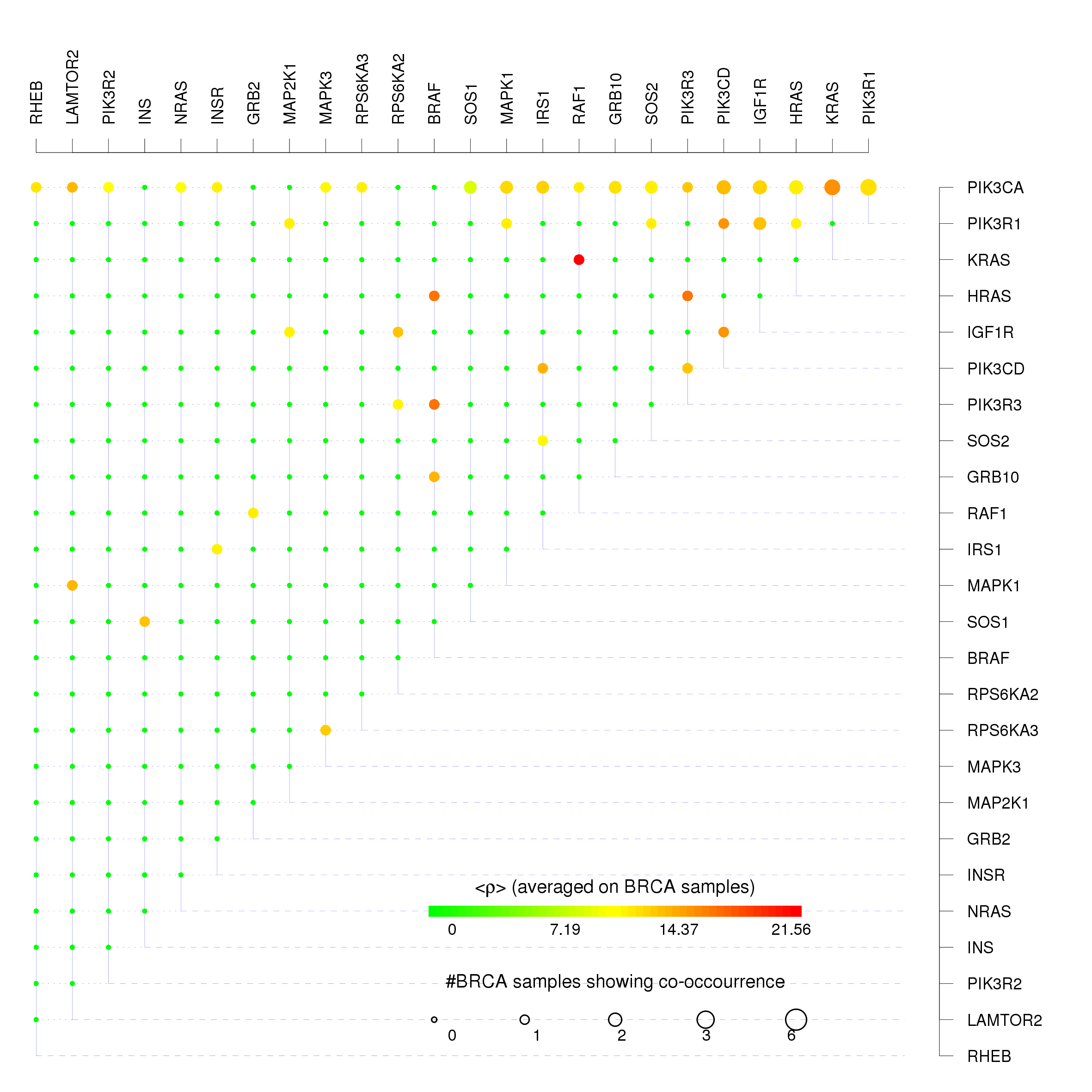
**

**Figure S8. Gene-gene co-occurrences and frailty measures for mTor signaling pathway.** For each couple of mTor signaling pathway co-mutated genes we show the average frailty < ρ > measured on all the BC pathway having the correspondent couple of genes simultaneously mutated using the color scale from green corresponding to null frailty (maximum resilience) to red corresponding to the maximum frailty found in real BC data. The size of each dot is proportional to the log-log scaled number of BC patients presenting the co-mutation.

**Supplementary Figure S9**

**
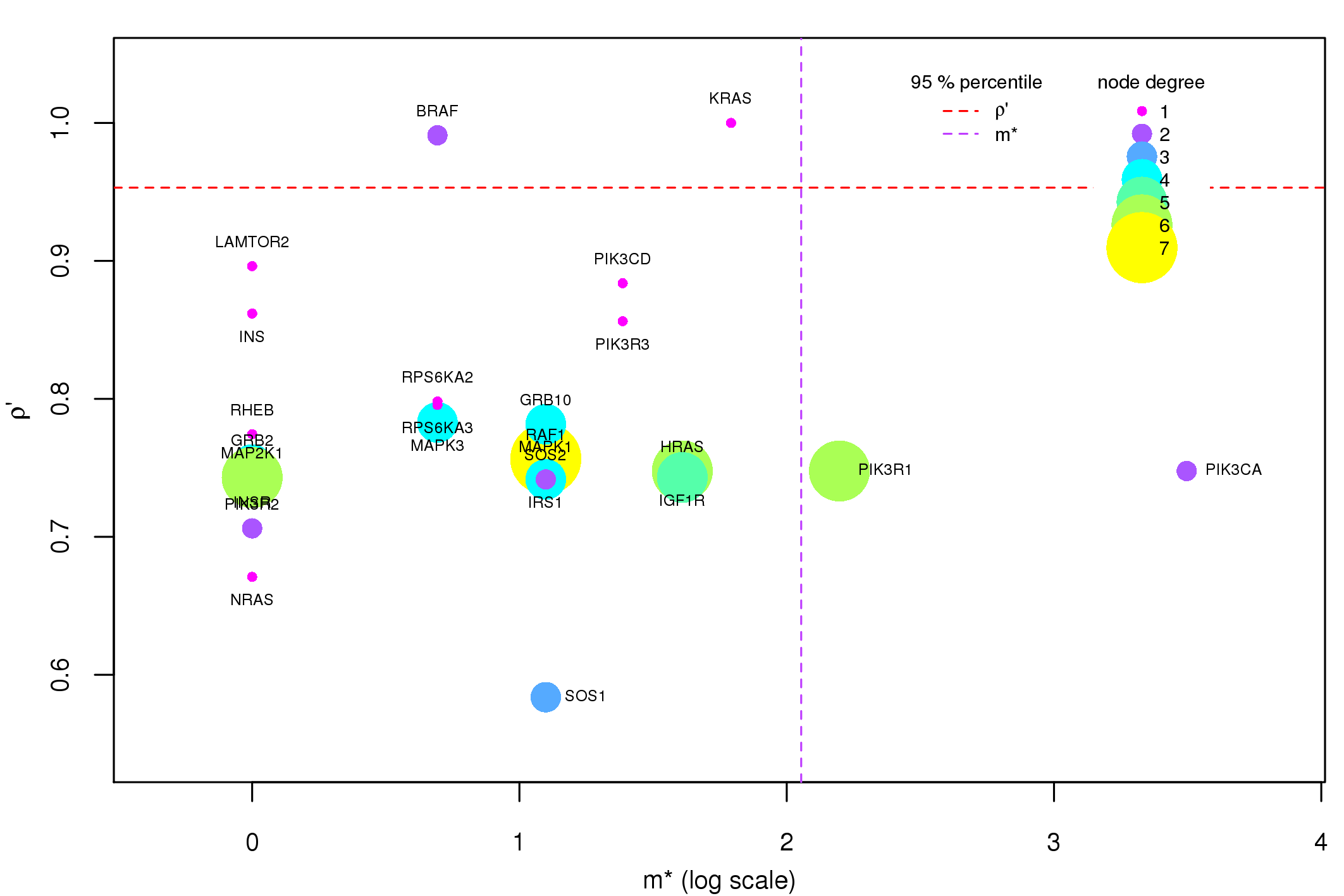
**

**Figure S9. Components of the GFI for mTor signaling pathway**. On the mTor signaling pathway gene network we plot the GFI components for each gene of the network that has at least one non-null component. The x-axis is an elementary transformation of the gene co-occurrence σ (*m*∗ = σ · *nsamples* ) and it is displayed in log scale for visualization issues.

**------------------------- PI3K-Akt signaling pathway --------------------------**

**Supplementary Figure S10**

**
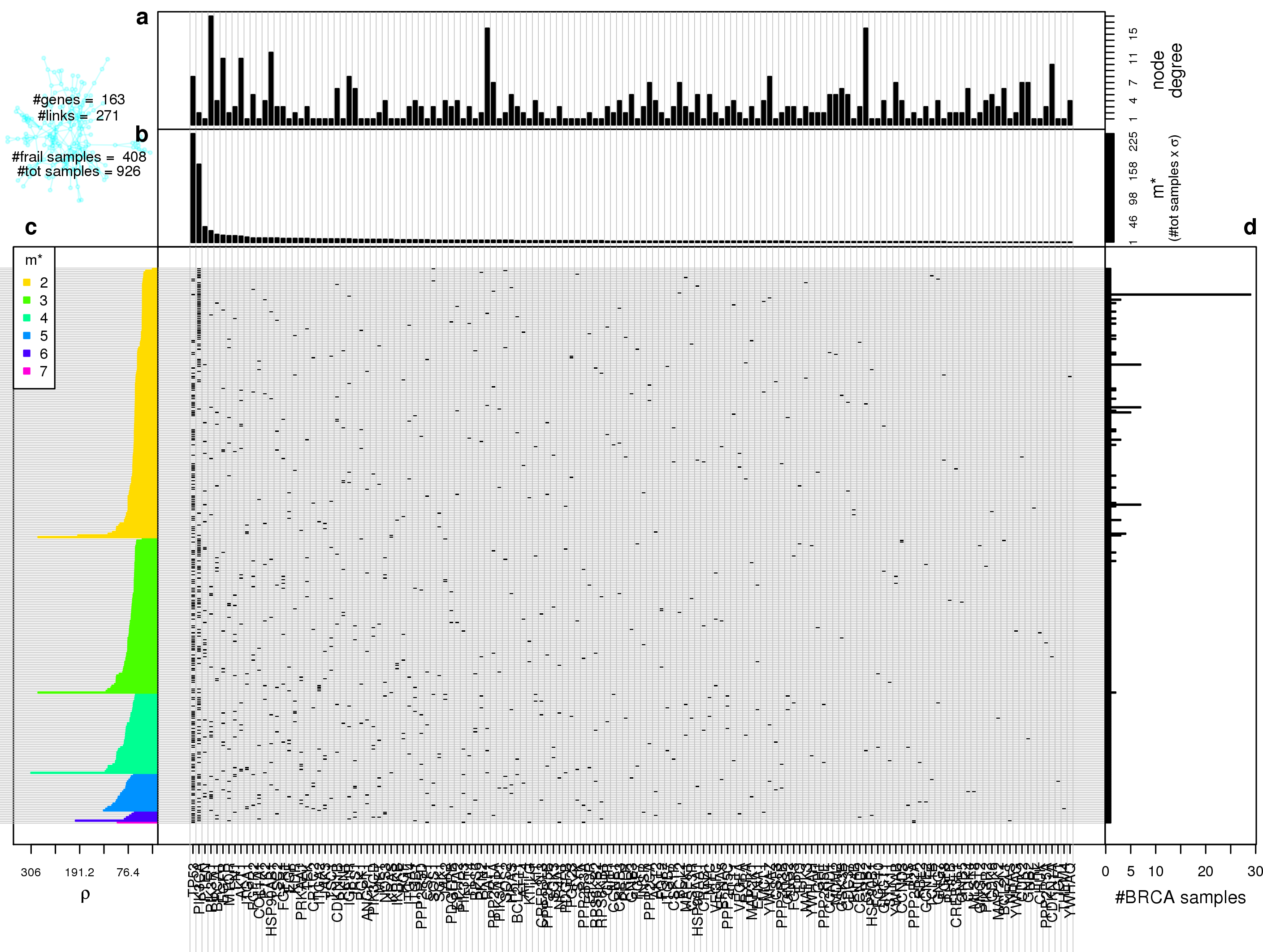
**

**Figure S10. Gene network frailty analysis for PI3K-Akt signaling pathway.** Central plot: in each row we plot the somatic mutations co-occurring in the same samples on the PI3K-Akt signaling pathway gene network. Rows are ordered as the left-hand side plot, columns are sorted from left to right for decreasing *m*∗. Top panels: from top to bottom is respectively displayed each gene node degree in the target network (a) and the number of patients in which each gene is mutated together with at least another gene (*m*∗) (b); the columns of the central plot are sorted according to decreasing *m*∗. All results were obtained using BC data mapped on KEGG’s PI3K-Akt signaling pathway gene network. Left-hand side plot (c): Gene network frailty is plotted for each SM configuration; the ordering on the y-axis is determined in first instance by number of mutations per patient occurring on the gene network, and then in each class the configurations are sorted by ρ. Right-hand side plot (d): for each row of the central plot is displayed the number of patients presenting the corresponding SM configuration.

**Supplementary Figure S11**

**
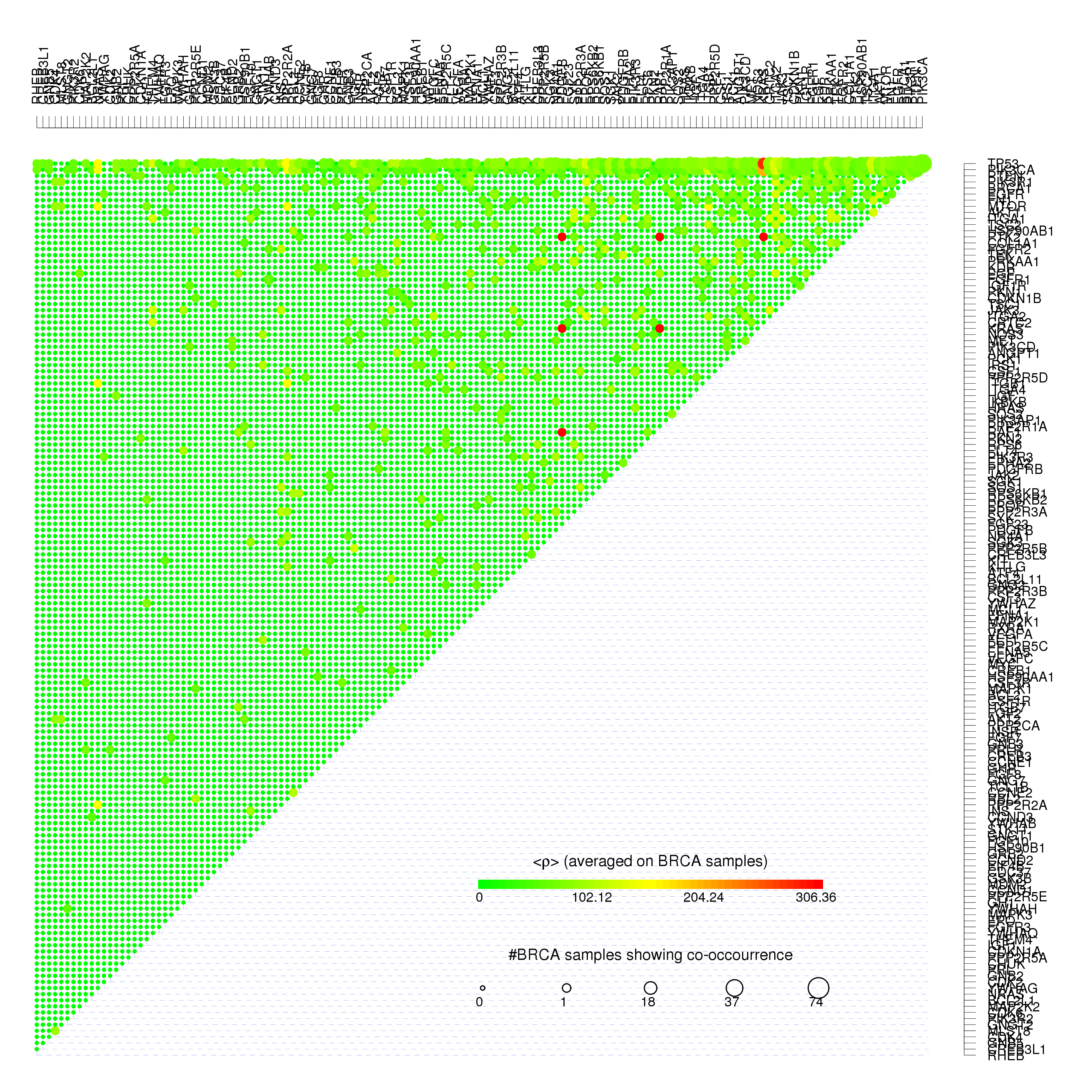
**

**Figure S11. Gene-gene co-occurrences and frailty measures for PI3K-Akt signaling pathway.** For each couple of PI3K-Akt signaling pathway co-mutated genes we show the average frailty < ρ > measured on all the BC pathway having the correspondent couple of genes simultaneously mutated using the color scale from green corresponding to null frailty (maximum resilience) to red corresponding to the maximum frailty found in real BC data. The size of each dot is proportional to the log-log scaled number of BC patients presenting the co-mutation.

**Supplementary Figure S12**

**
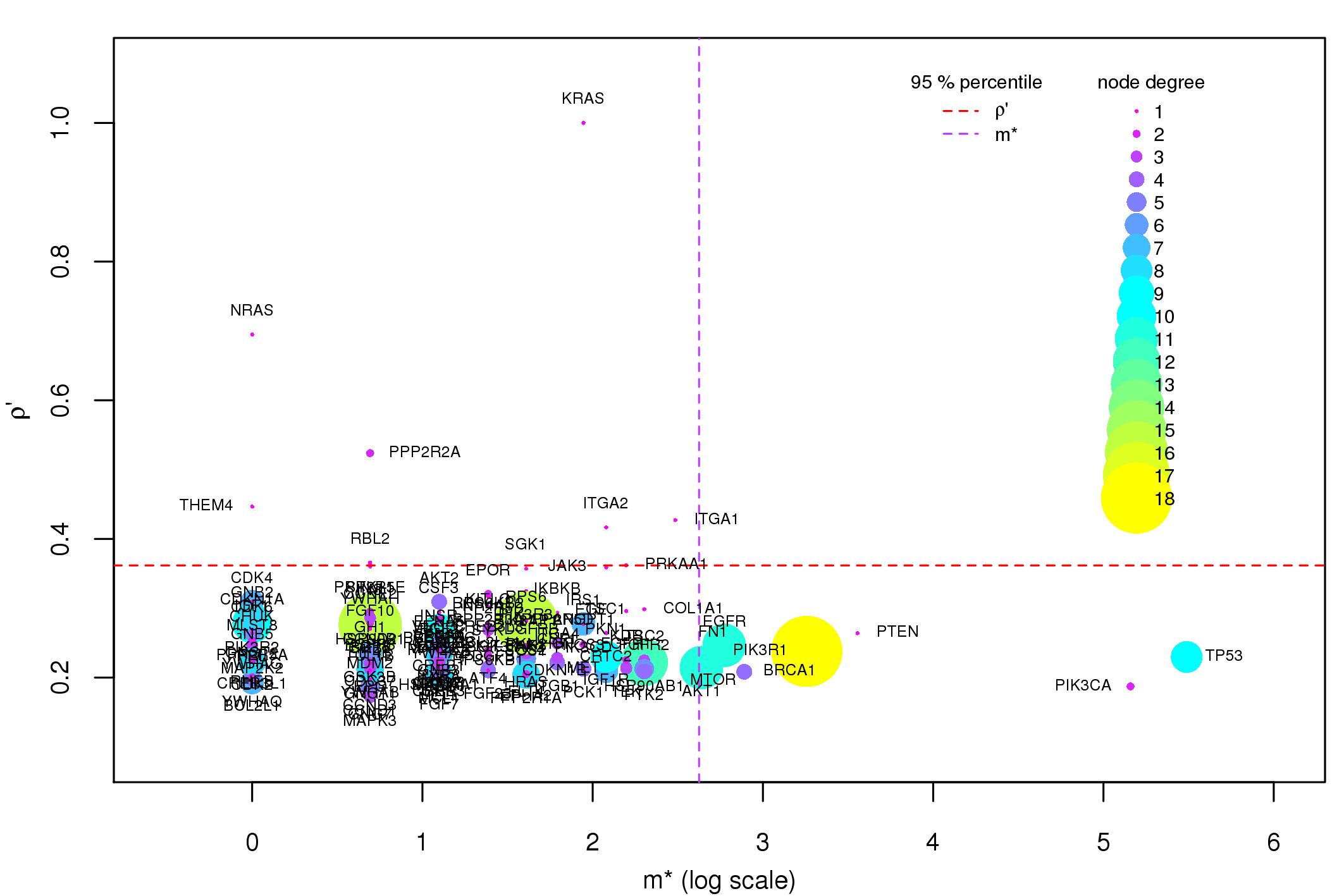
**

**Figure S12. Components of the GFI for PI3K-Akt signaling pathway**. On the PI3K-Akt signaling pathway gene network we plot the GFI components for each gene of the network that has at least one non-null component. The x-axis is an elementary transformation of the gene co-occurrence σ (*m*∗ = σ · *nsamples* ) and it is displayed in log scale for visualization issues.

**------------------------- Rap1 signaling pathway --------------------------------**

**Supplementary Figure S13**

**
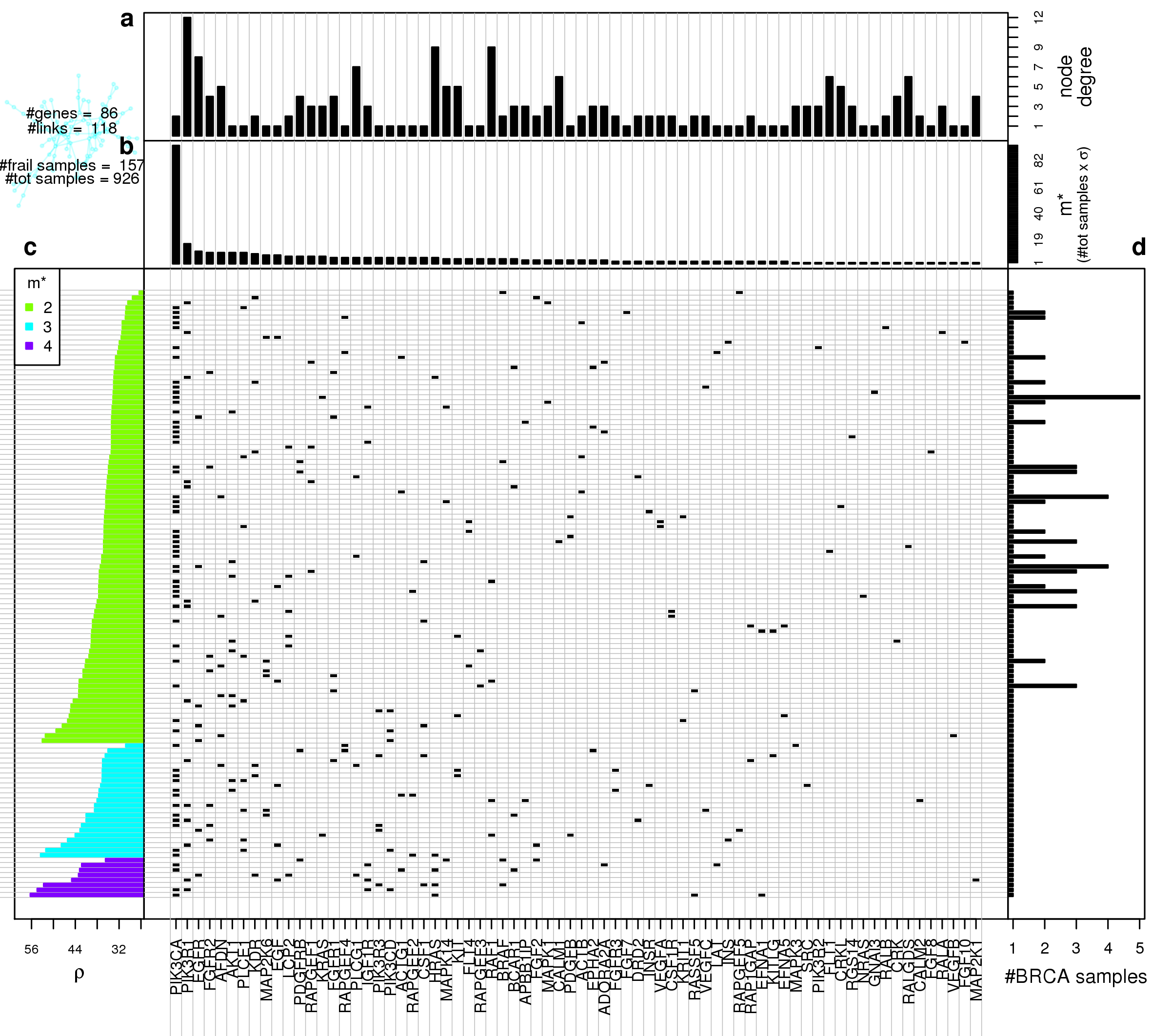
**

**Figure S13. Gene network frailty analysis for Rap1 signaling pathway.** Central plot: in each row we plot the somatic mutations co-occurring in the same samples on the Rap1 signaling pathway gene network. Rows are ordered as the left-hand side plot, columns are sorted from left to right for decreasing *m*∗. Top panels: from top to bottom is respectively displayed each gene node degree in the target network (a) and the number of patients in which each gene is mutated together with at least another gene (*m*∗) (b); the columns of the central plot are sorted according to decreasing *m*∗. All results were obtained using BC data mapped on KEGG’s Rap1 signaling pathway gene network. Left-hand side plot (c): Gene network frailty is plotted for each SM configuration; the ordering on the y-axis is determined in first instance by number of mutations per patient occurring on the gene network, and then in each class the configurations are sorted by ρ. Right-hand side plot (d): for each row of the central plot is displayed the number of patients presenting the corresponding SM configuration.

**Supplementary Figure S14**

**
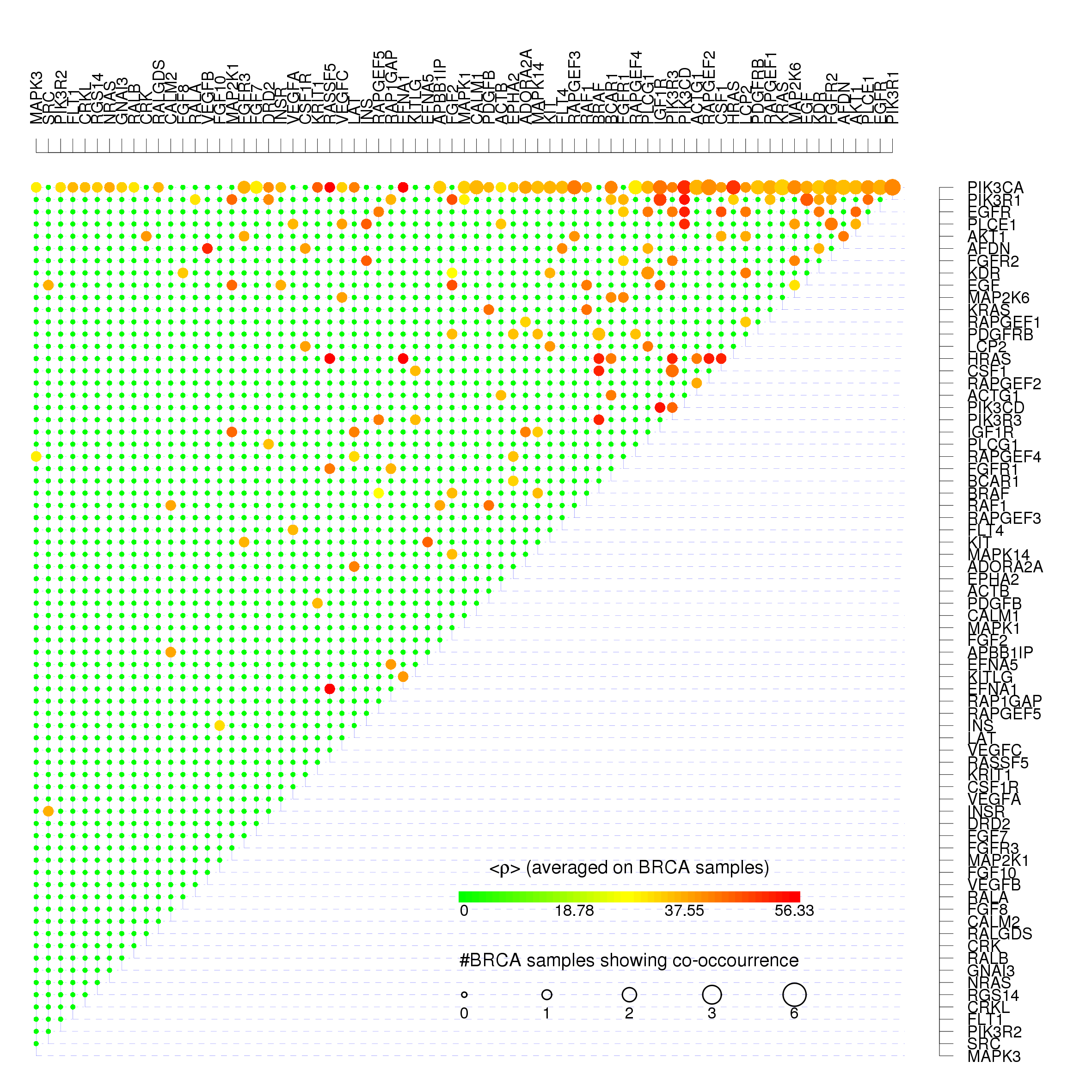
**

**Figure S14. Gene-gene co-occurrences and frailty measures for Rap1 signaling pathway.** For each couple of Rap1 signaling pathway co-mutated genes we show the average frailty < ρ > measured on all the BC pathway having the correspondent couple of genes simultaneously mutated using the color scale from green corresponding to null frailty (maximum resilience) to red corresponding to the maximum frailty found in real BC data. The size of each dot is proportional to the log-log scaled number of BC patients presenting the co-mutation.

**Supplementary Figure S15**

**
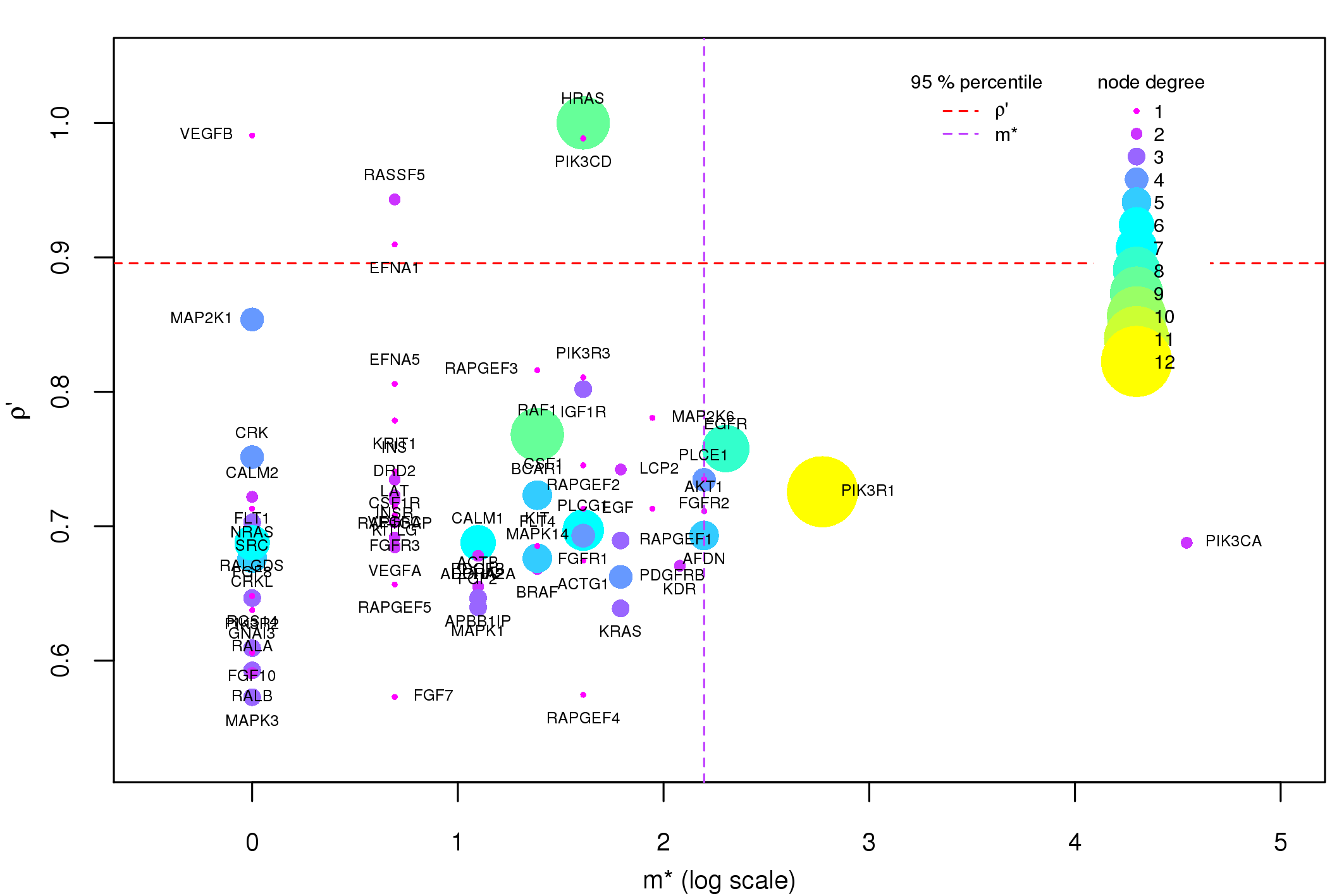
**

**Figure S15. Components of the GFI for Rap1 signaling pathway**. On the Rap1 signaling pathway gene network we plot the GFI components for each gene of the network that has at least one non-null component. The x-axis is an elementary transformation of the gene co-occurrence σ (*m*∗ = σ · *nsamples* ) and it is displayed in log scale for visualization issues.
